# Author Correction: Cancer-associated Histone H3 N-terminal arginine mutations disrupt PRC2 activity and impair differentiation

**DOI:** 10.1038/s41467-024-53502-z

**Published:** 2024-10-22

**Authors:** Benjamin A. Nacev, Yakshi Dabas, Matthew R. Paul, Christian Pacheco, Michelle Mitchener, Yekaterina Perez, Yan Fang, Alexey A. Soshnev, Douglas Barrows, Thomas Carroll, Nicholas D. Socci, Samantha C. St. Jean, Sagarika Tiwari, Michael J. Gruss, Sebastien Monette, William D. Tap, Benjamin A. Garcia, Tom Muir, C. David Allis

**Affiliations:** 1 Department of Medicine, University of Pittsburgh School of Medicine, Pittsburgh, PA 15213 USA; 2 Department of Pathology, University of Pittsburgh School of Medicine, Pittsburgh, PA 15213 USA; 3 UPMC Hillman Cancer Center, Pittsburgh, PA 15213 USA; 4 Laboratory of Chromatin Biology and Epigenetics, The Rockefeller University, New York, NY 10065 USA; 5 Bioinformatics Resource Center, The Rockefeller University, New York, NY 10065 USA; 6 Department of Chemistry, Princeton University, Princeton, NJ 08544 USA; 7 Department of Biochemistry and Biophysics, Perelman School of Medicine, University of Pennsylvania, Philadelphia, PA 19104 USA; 8 Department of Medicine, Memorial Sloan Kettering Cancer Center, New York, NY 10065 USA; 9 Bioinformatics Core, Memorial Sloan Kettering Cancer Center, New York, NY 10065 USA; 10 Laboratory of Comparative Pathology, Memorial Sloan Kettering Cancer Center, New York, NY 10065 USA; 11 Department of Biochemistry and Molecular Biophysics, Washington University School of Medicine, St. Louis, MO 63110 USA; 12 Present Address: Department of Neuroscience, Developmental and Regenerative Biology, The University of Texas at San Antonio, San Antonio, TX 78249 USA

**Keywords:** Epigenetics, Cancer epigenetics, Cancer genomics, Sarcoma, Embryonic stem cells

Correction to: *Nature Communications* 10.1038/s41467-024-49486-5, published online 17 June 2024

The Source Data associated with this Article contained an error in the source data spreadsheet for Fig. 5D, in which one value changed from 66.81 to 67.11 and another from 60.99 to 61.07 during the final revision. The correct version of the Source Data can be found as Source Data associated with this Correction. The Source Data associated with the original article has been replaced with the correct file.

## Source data

Revised Source data

