# Supplementary material for: Cancer-associated Histone H3 N-terminal arginine mutations disrupt PRC2 activity and impair differentiation
Source: Nat Commun. 2024 Jun 17;15:5155. doi: 10.1038/s41467-024-49486-5 (PMC11183192; doi:10.1038/s41467-024-49486-5)
Supplement: Supplementary file 1 — Supplementary Information [file 41467_2024_49486_MOESM1_ESM.pdf]

Supplementary Figure 1

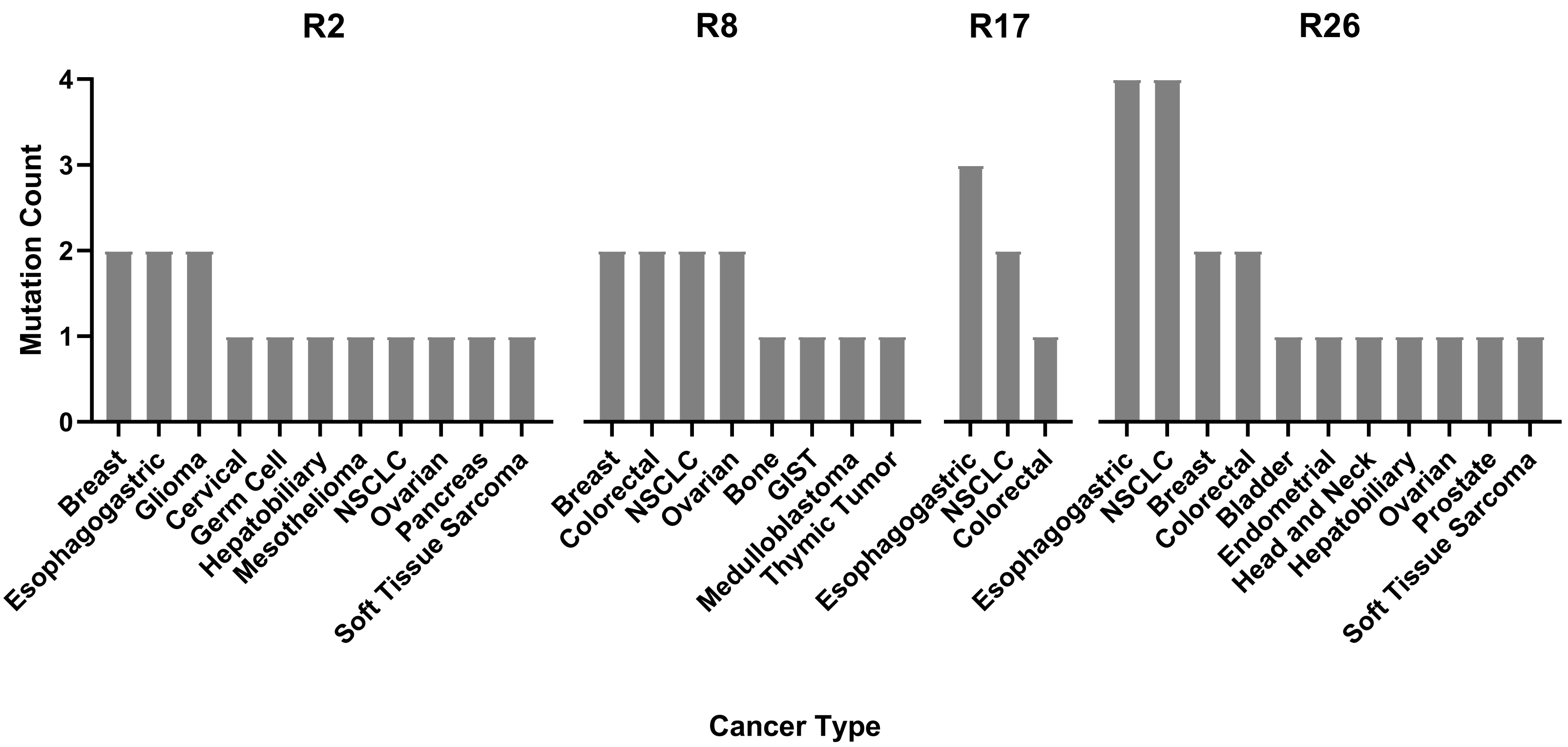

Supplementary Fig. 1. H3 N-terminal missense mutations at arginine residues by tumor type. Count of mutations per "Curated_Main_Cancer_Type" from Supplementary Table 1 are plotted and grouped for each arginine position.

Supplementary Figure 2

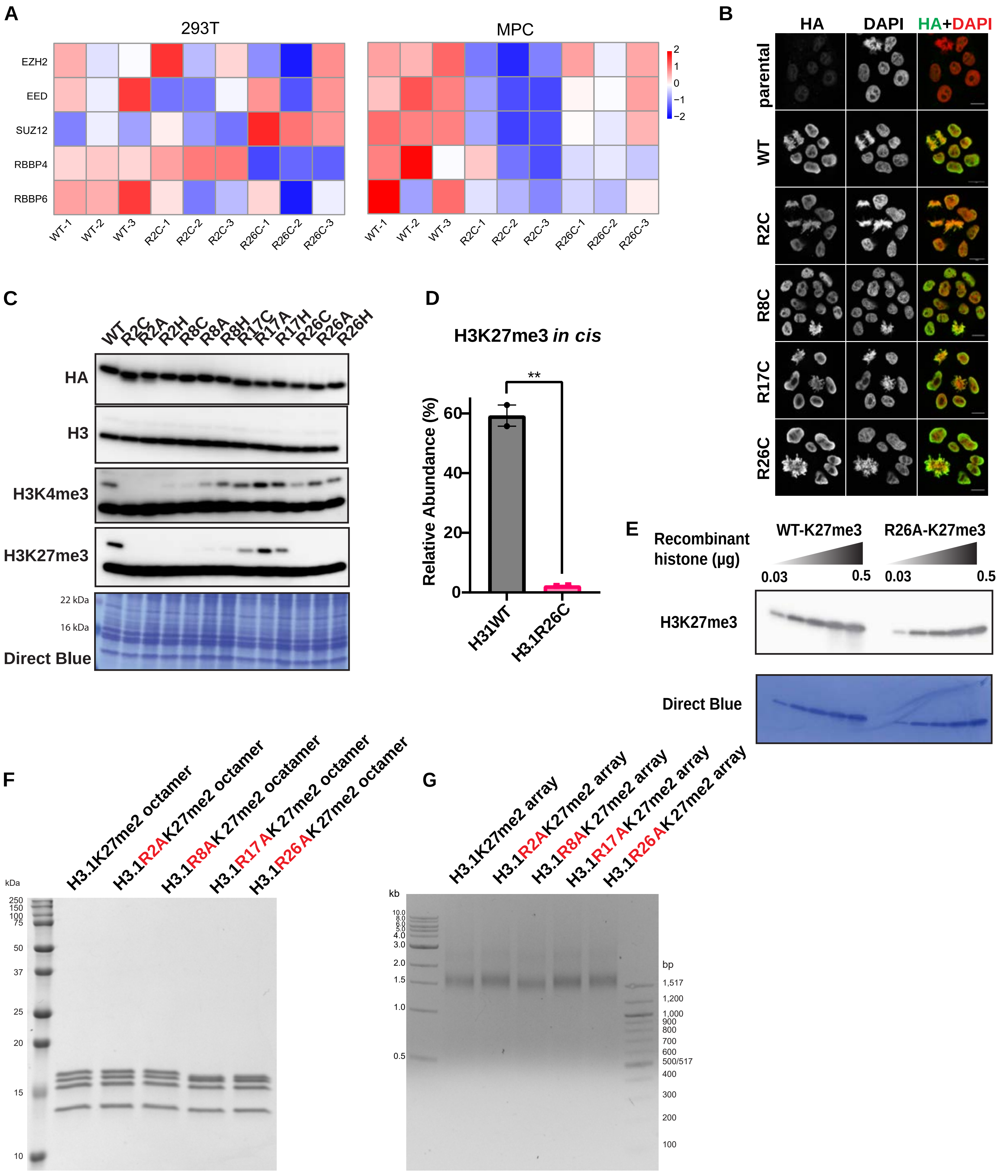

Supplementary Fig. 2. Characterization of H3 arginine mutations and nucleosome array assembly. A Expression of core PRC2 complex members. Color gradient denotes the z-score of rlog(counts). n = 3 independent replicates each individually shown and denoted by the sample suffix "-n". **B** Immunofluorescence imaging of H3WT- or H3 arginine mutant-expressing HEK293T cells. Anti-HA and DAPI signals co-localize to condensed, mitotic chromatin. Bars = 10 µm. Representative images from one experiment performed in parallel for all groups. **C** Immunoblot analysis comparing total H3, HA-epitope, or specific histone PTMs between H3WT for different H3 arginine missense mutations across multiple arginine residues. Blots are representative of two independent experiments. kDa, kilodalton. **D** Middle-down mass spectrometry was used to determine the relative abundance of H3K27me3 in H3WT and H3R26C mutant histone tails. n = 2 independent experiments. Group means were compared using an unpaired two-sided t-test; t=16.04, df=2, 95% CI (-72.23, -41.67), R^2=0.9923, **, p=0.0039. Error bars are standard error of the mean. Source data are provided as a Source Data file. **E** Anti-H3K27me3 antibody recognition of the mark in semisynthetic H3WT-K27me3 and H3R26A-K27me3 histones. Data are representative of one blot using using these exact conditions. **F** SDS-PAGE analysis of purified octamers containing semisynthetic wild-type or arginine mutant H3.1K27me2, visualized by Coomassie staining. Data are representative of one blot. The molecular weight ladder is shown on the left with mass noted in kilodaltons (kDa). **G** Native PAGE analysis of wild-type and arginine mutant H3.1K27me2 12-mer arrays (DNA visualized with ethidium bromide staining). The left ladder notes DNA fragment size in kilobase pairs (kb) and the right ladder notes sizes in base pairs (bp). Data are representative of one blot.

Supplementary Figure 3

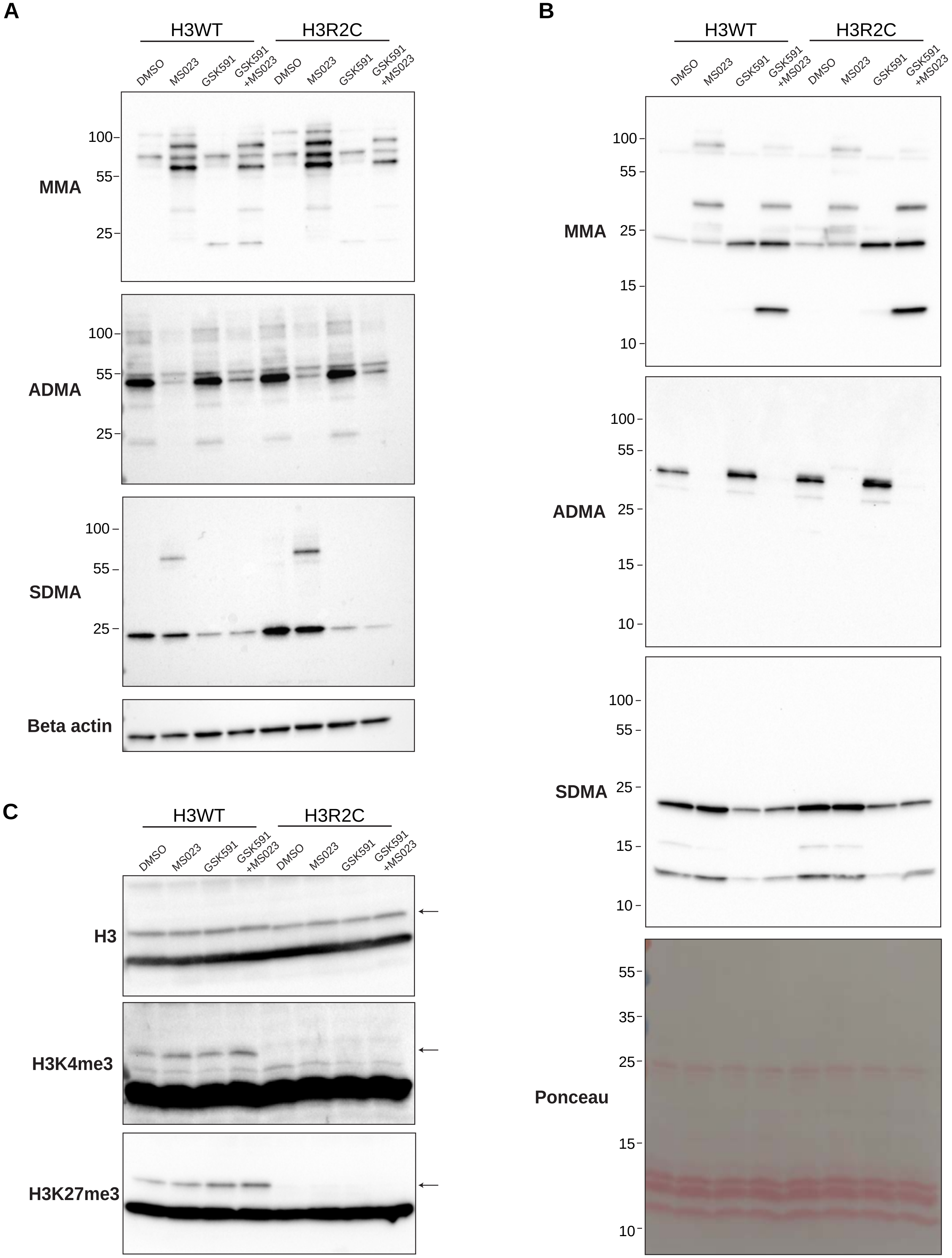

Supplementary Fig. 3. Inhibition of arginine methyltransferases does not affect H3K4me3 or H3K27me3. A Immunoblots demonstrating the effect of a 2-day treatment with MS023 (300 nM), GSK591 (300 nM), or GSK591 (300 nM) + MS023 (300 nM), or vehicle control (0.5% DMSO) on monomethyl arginine (MMA), asymmetric dimethylarginine (ASDM) or symmetric dimethylarginine (SDMA) in whole cell lysates from H3WT or H3R2C expressing MPCs. Beta actin is shown as a loading control using the same samples, but run on a separate blot. Positions of molecular weight markers (kDa) are shown on the left of the blots. **B** Histone acid extracts from cells treated as in (A) were analyzed by immunoblot using methylarginine antibodies or stained with ponceau. Positions of prestained molecular weight markers (kDa) are shown on the left of the blots. **C** Immunoblots of histone extracts from cells treated as in (A) and (B) and probed for total H3, H3K4me3, or H3K27me3. Arrows indicate the transgenic mutant epitope-tagged histone. All blots are representative of two independent experiments.

Supplementary Figure 4

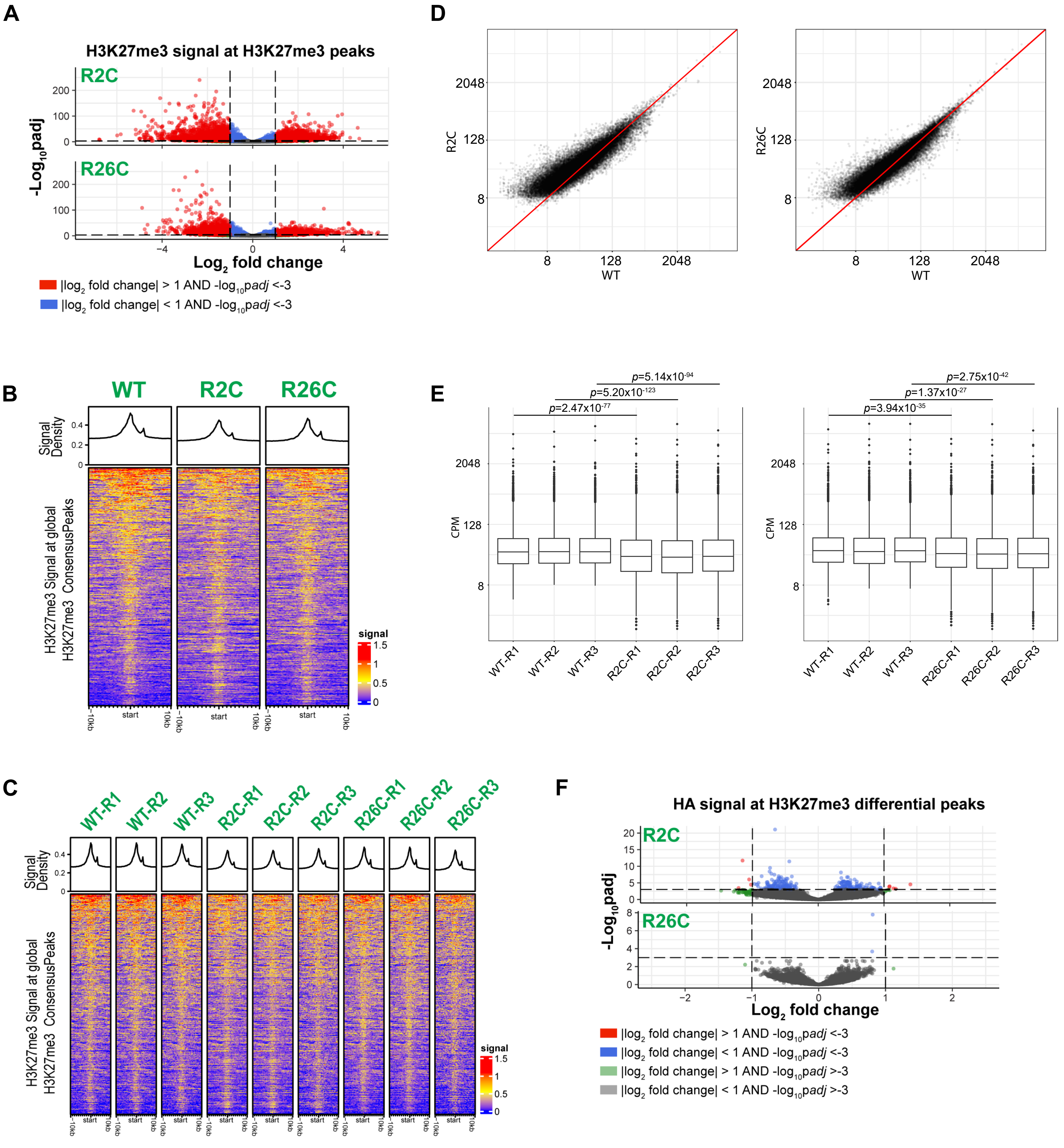

Supplementary Fig. 4. H3K27me3 and HA differential peak analysis (H3R2C or H3R26C versus H3WT). A Volcano plots of H3K27me3 signal at H3K27me3 consensus peaks for H3R2C versus H3WT and H3R26C versus H3WT (n=3 biological replicates). **B** H3K27me3 signal at H3K27me3 consensus peaks based on mean values (n=3 biological replicates) and for **C** individual replicates, each denoted by sample the suffix “-Rn”. **D** Scatter plots comparing H3K27me3 signal at all peaks. Counts per million (CPM) shown on $\log_2$ scale axes. **E** Boxplots representing data from (D) for individual replicates, each denoted by sample the suffix “-Rn”. p-values are shown based on a two-sided Wilcoxon test. For the boxplots, the median is displayed by the internal line, the lower hinge represents the 25th percentile and the upper hinge represents the 75th percentile. The whiskers indicate 1.5 x the interquartile range, with points beyond the whiskers representing outliers. n=12,410 peaks for the H3R2C comparisons and n=11,563 peaks for the H3R26C comparisons. **F** Volcano plots of HA signal at H3K27me3 differential peaks in H3R2C versus H3WT and H3R26C versus H3WT (n=3 biologic replicates).

Supplementary Figure 5

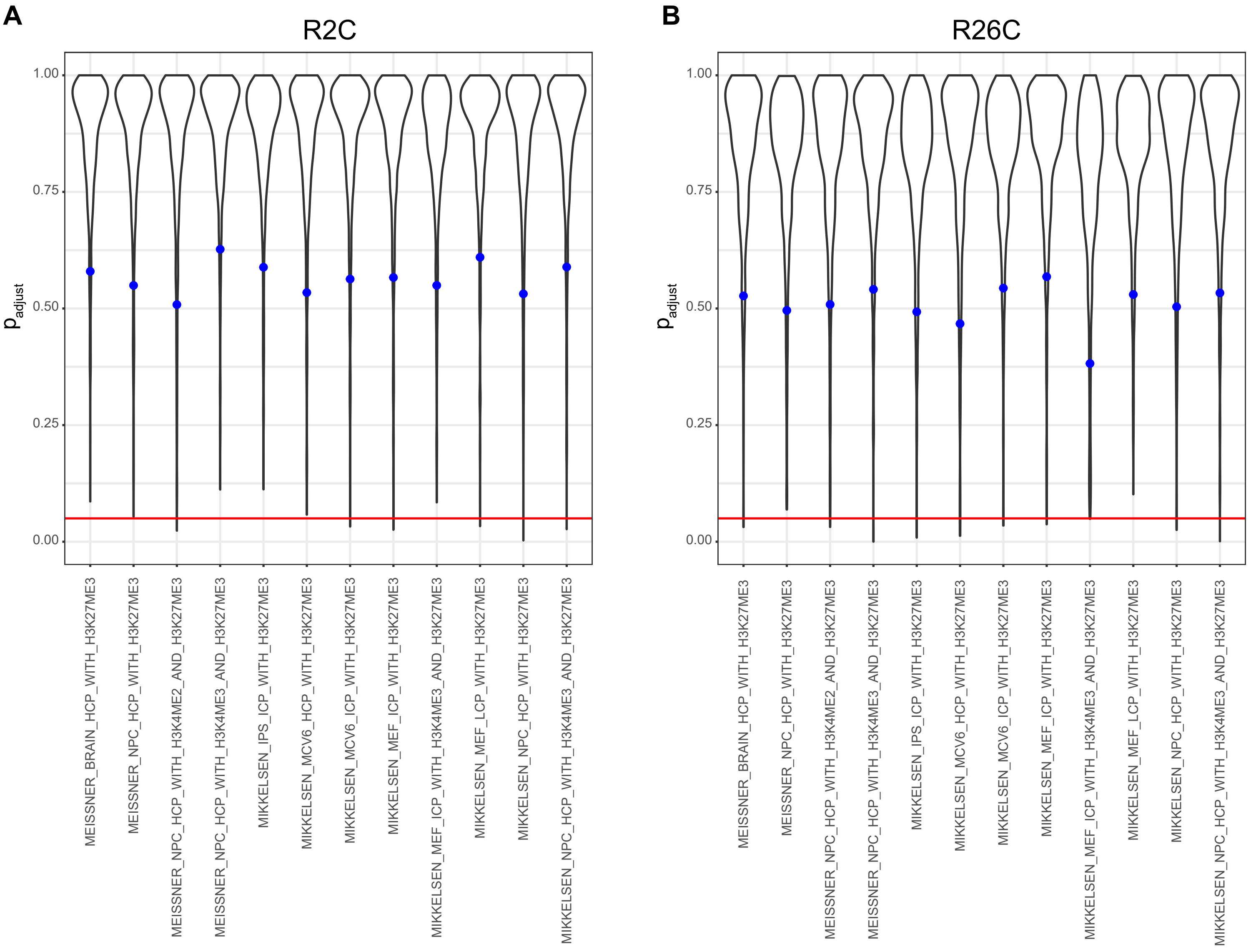

Supplementary Fig. 5. Randomly selected gene cohorts do not significantly overlap with polycomb genesets. GO term enrichment tests for 1,000 randomly permuted gene cohorts corresponding to **A** H3R2C (2114 genes) or **B** H3R26C (1277 genes). Blue points indicate the 95% percentile. Red line indicates padj=0.05.

Supplementary Figure 6

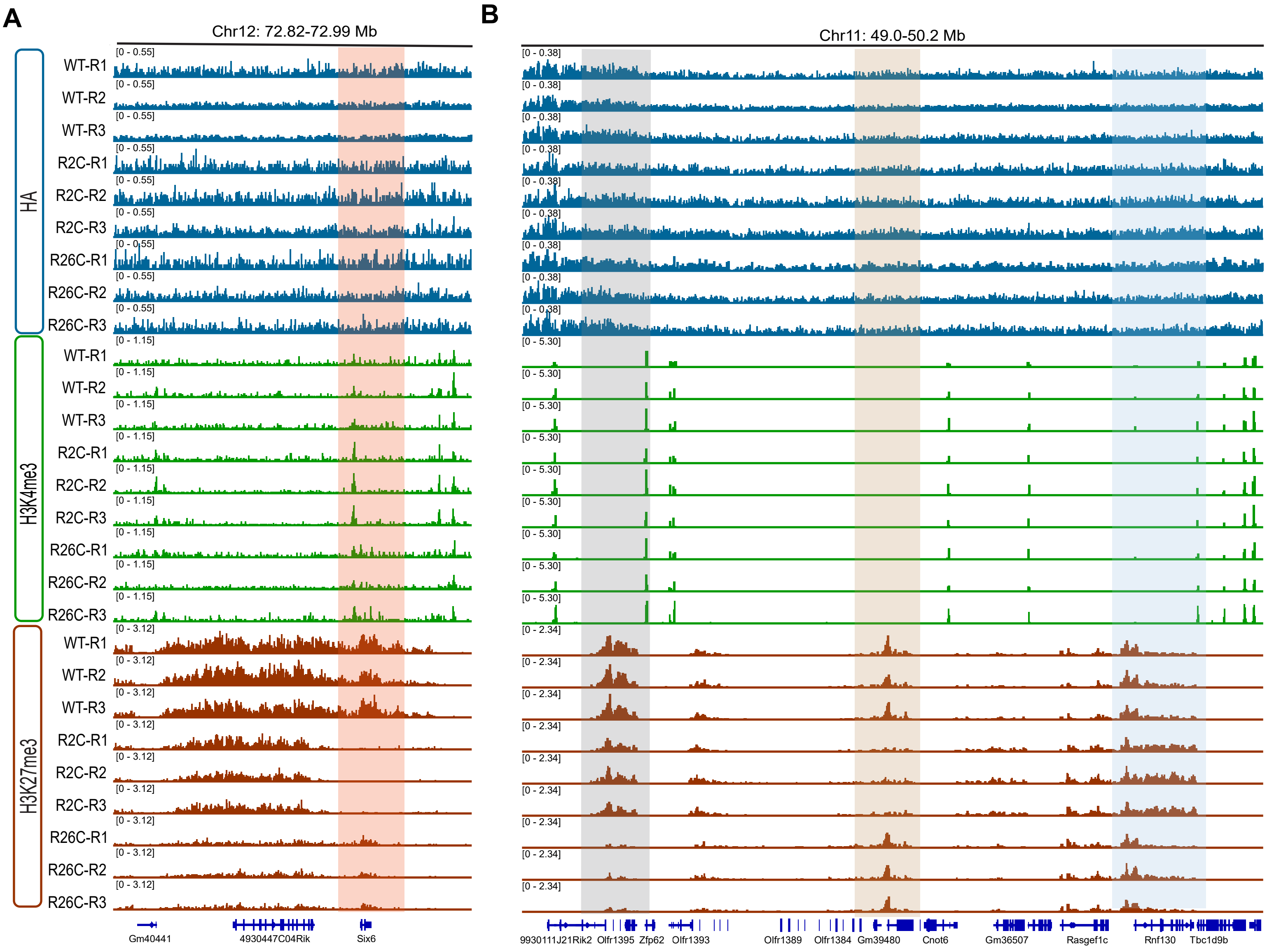

Supplementary Fig. 6. Regions of H3K27me3 peaks. IGV (version 2.15.4) tracks of representative regions with differential H3K27me3 peaks (mutant/WT) lost in **A** both H3R2C and HR26C (red shading), **B** lost in H3R2C alone (beige shading), H3R26C alone (grey shading) or gained in H3R2C (blue shading). Each biologic replicate (n=3) is individually shown. These data are the same as in Fig. 3D,E where replicates are overlayed.

Supplementary Figure 7

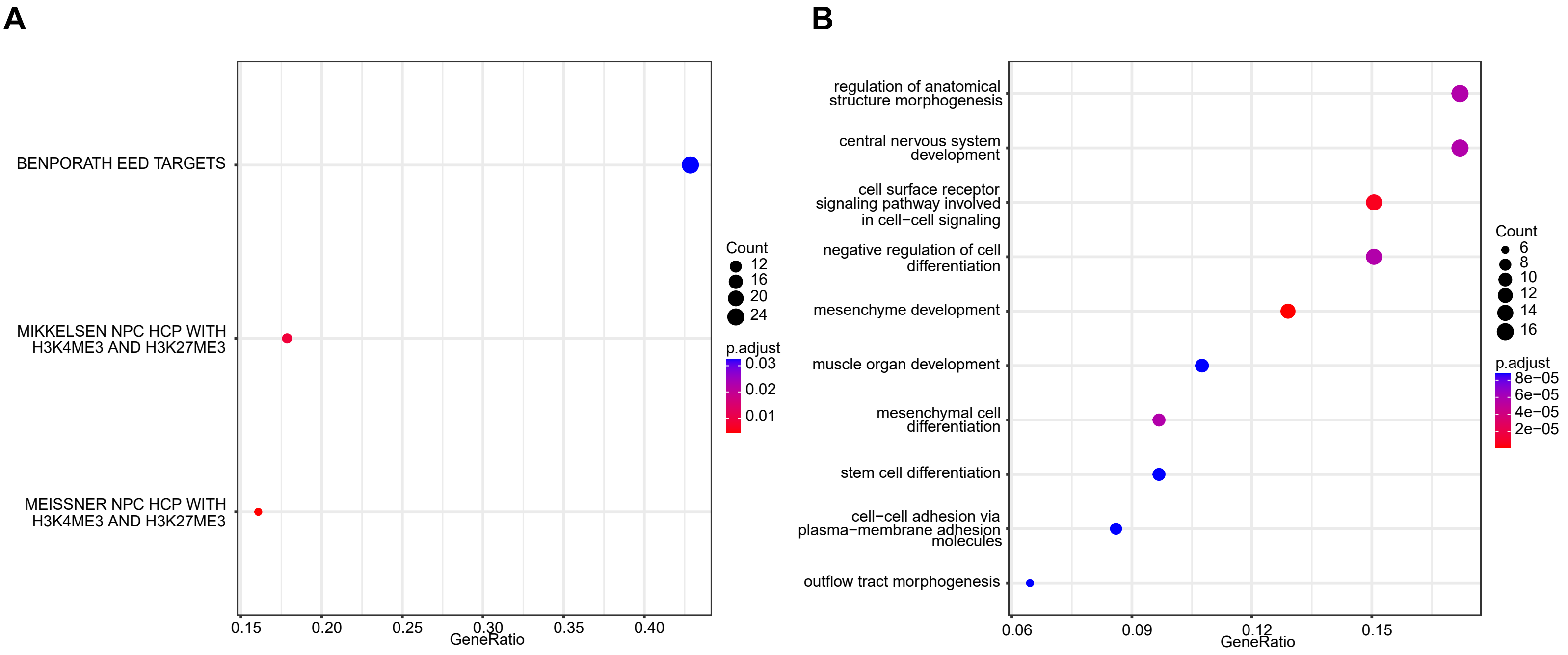

Supplementary Fig. 7. Pathways associated with overlapped H3K4me3 and H3K27me3 loss peaks (H3R2C versus H3WT). **A** Statistically significant overlap with Polycomb regulated gene sets. **B** Statistically significant overlap with GO terms (only a subset are shown, see Supplementary Table 6 for a complete list).

Supplementary Figure 8

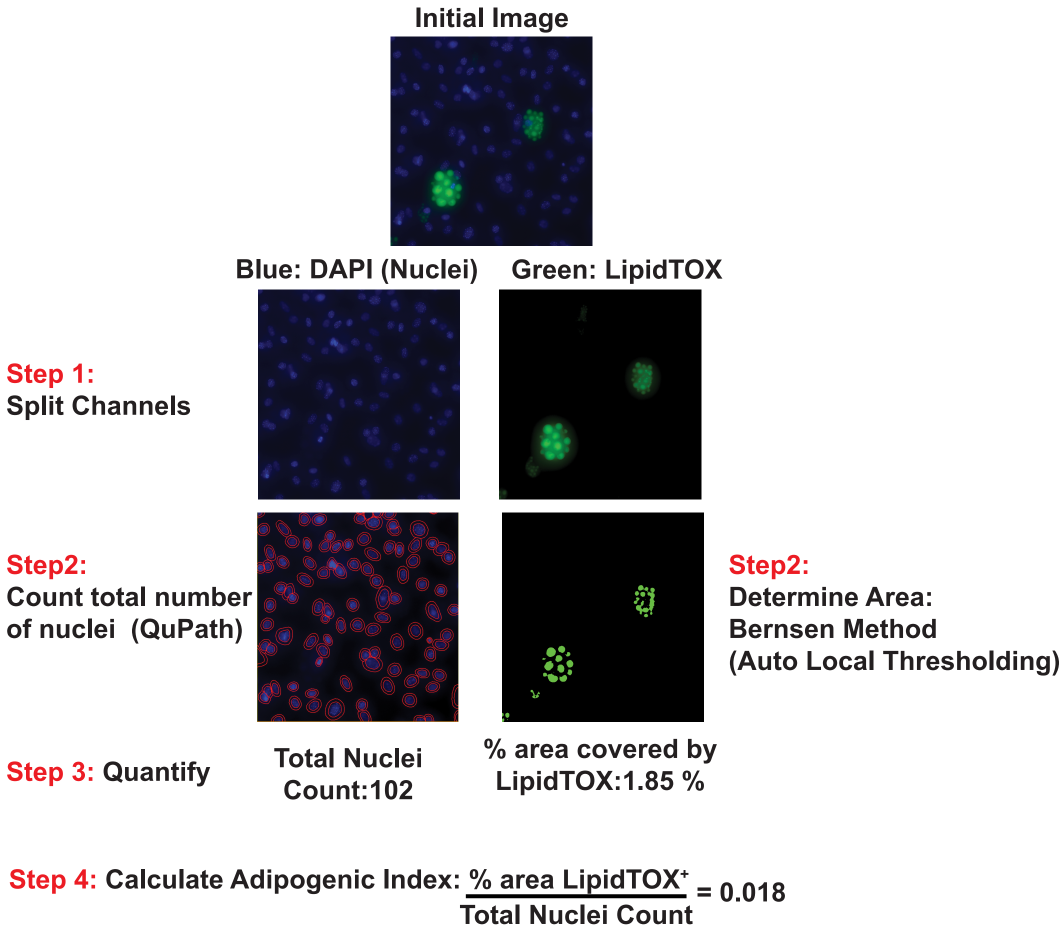

Supplementary Fig. 8. Adipogenic Index calculation schematic. Step 1: images were split into DAPI and LipidTOX channels. Step 2: In each of four non-overlapping images from the entire slide, the total number of nuclei were determined using QuPath and the area covered by LipidTOX was determined (Bernsen method of auto-local thresholding). Step 3: Nuclei counts and LipidTOX area were summed over all sub-images. Step 4: The Adipogenic Index was calculated based on the (percentage of total area positive for LipidTOX)/total nuclei count.

Supplementary Figure 9

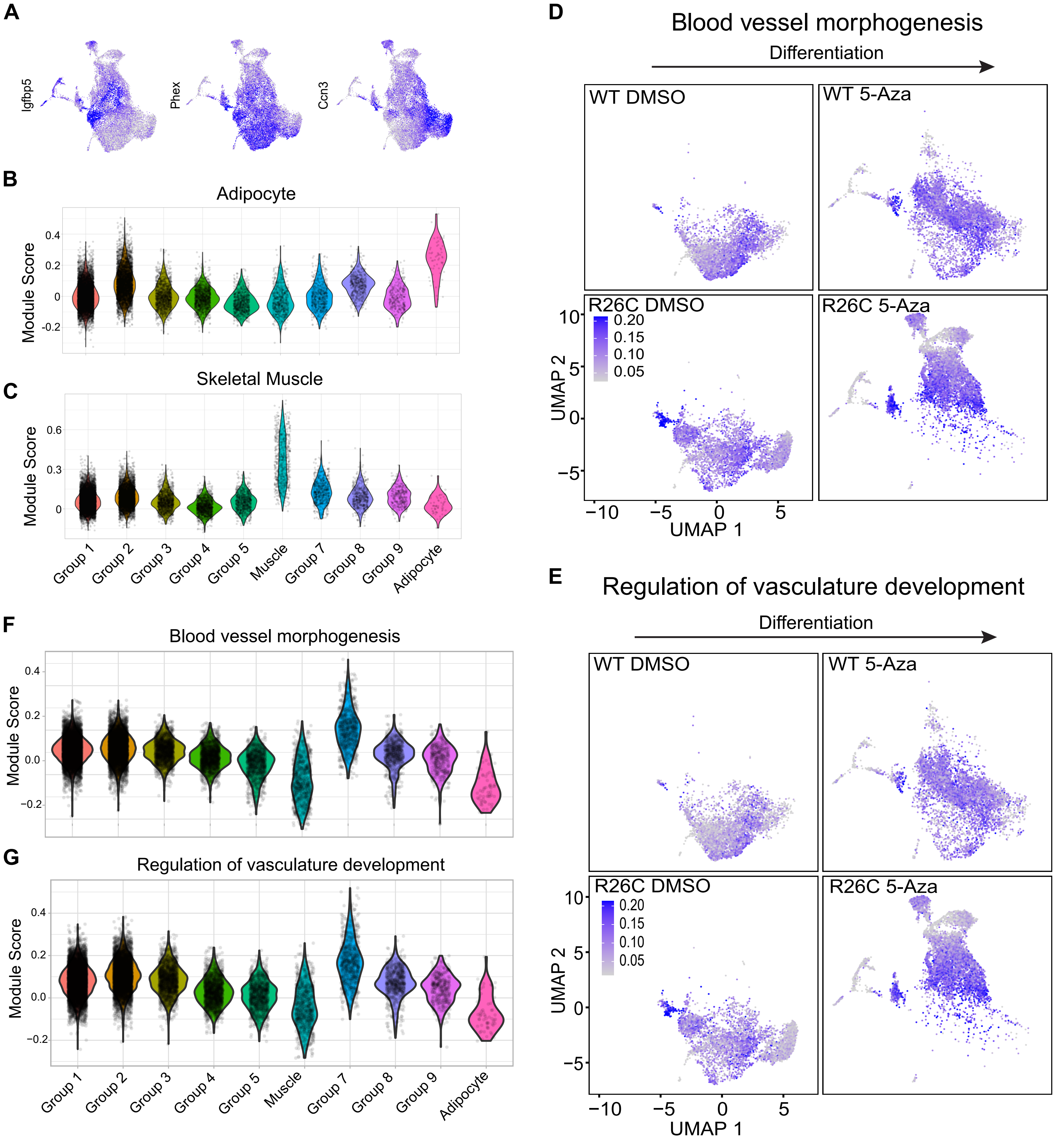

Supplementary Fig. 9: Cluster annotation and correlation with bulk RNA-seq data. A Expression of marker genes not included in Fig. 6. **B** Module scores for adipocyte and **C** skeletal muscle based on published gene sets. **D** Sample-specific feature plots based differential genes from bulk RNA-seq analysis in the GO term ‘Blood vessel morphogenesis’ or **E** ‘Regulation of vascular development’. **F** Group-specific module scores derived from the same gene list as in D, or **G** from the same gene list as in E.

Source Data:

Figure S2C

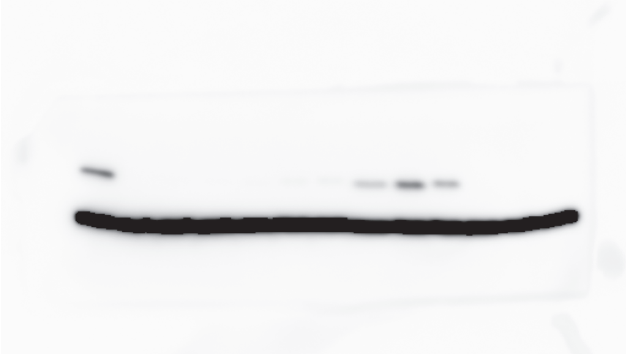

H3K27me3

~16 kDa

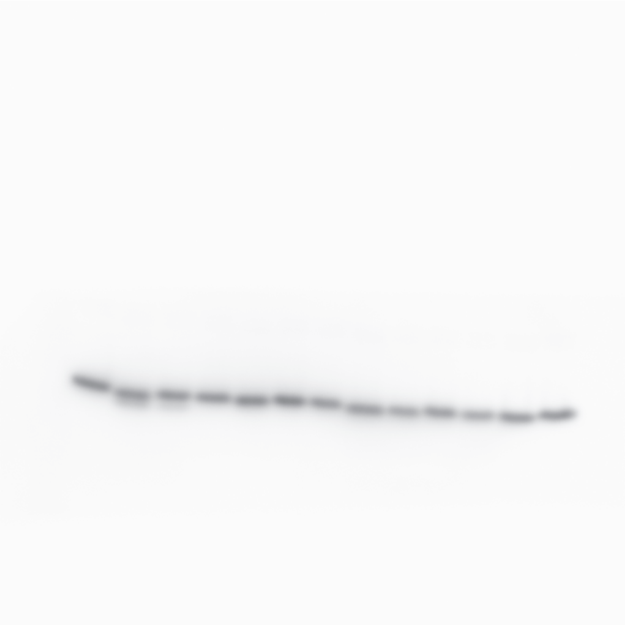

HA

~22 kDa

22 kDa

16 kDa

6 kDa

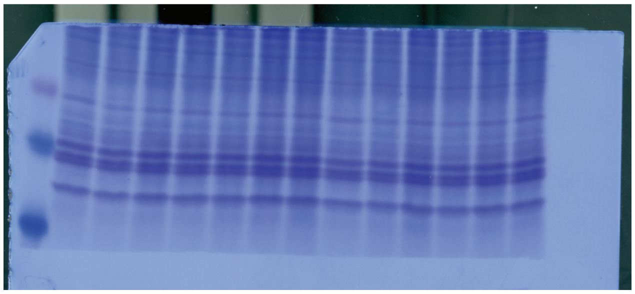

Direct Blue

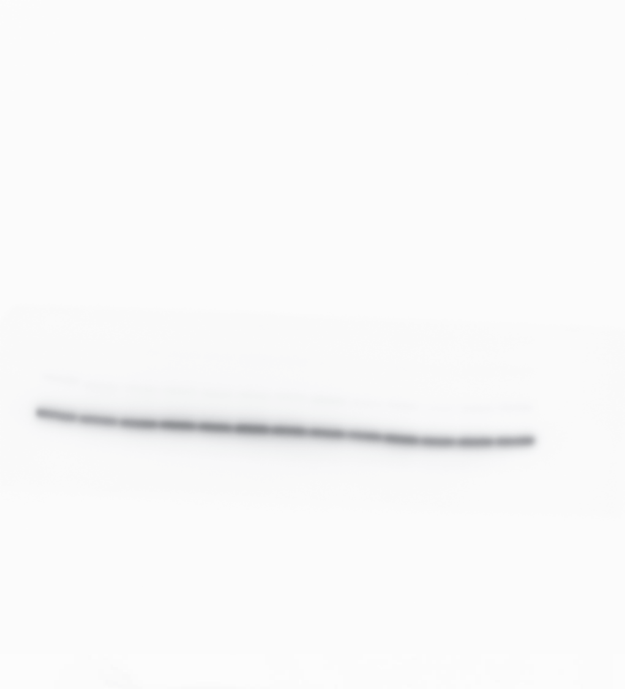

H3

~16 kDa

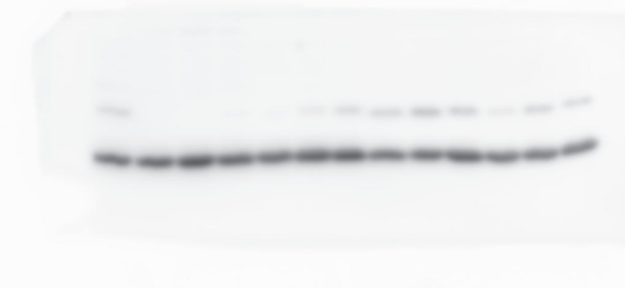

H3K4me3

~16 kDa

Figure S2E

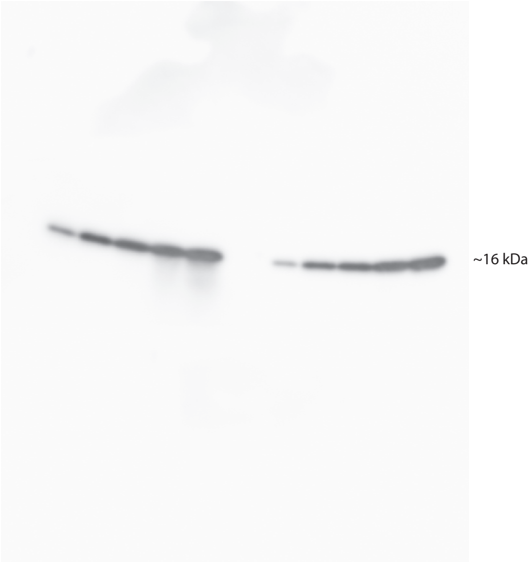

H3K27me3

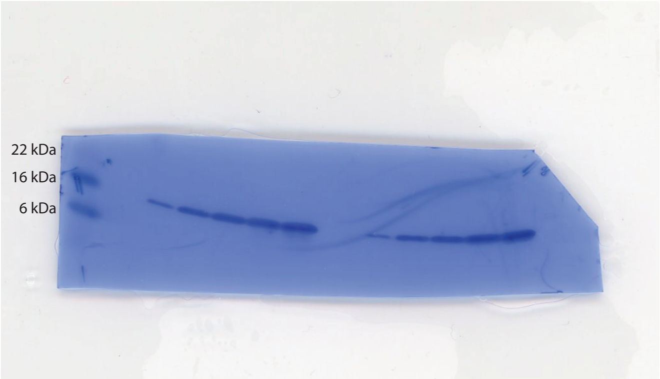

Direct Blue

Figure S3A

MMA

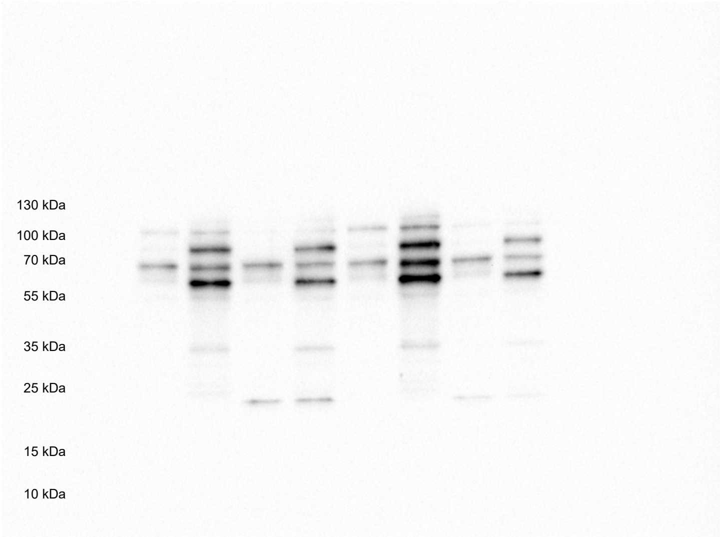

Beta-actin

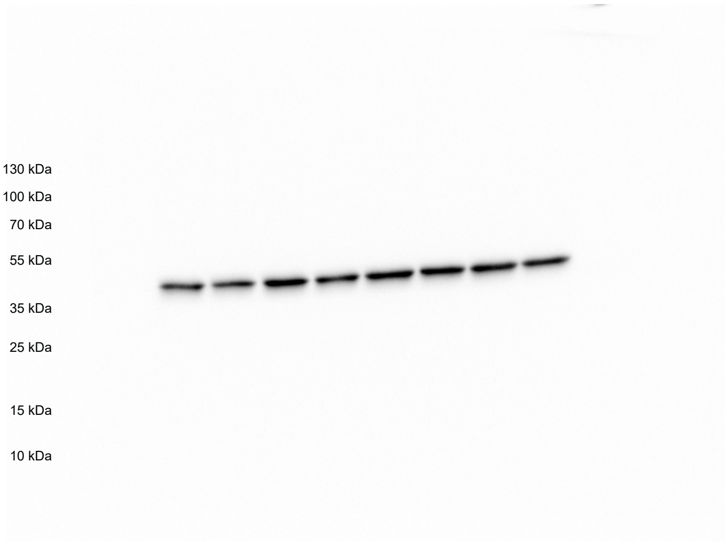

ADMA

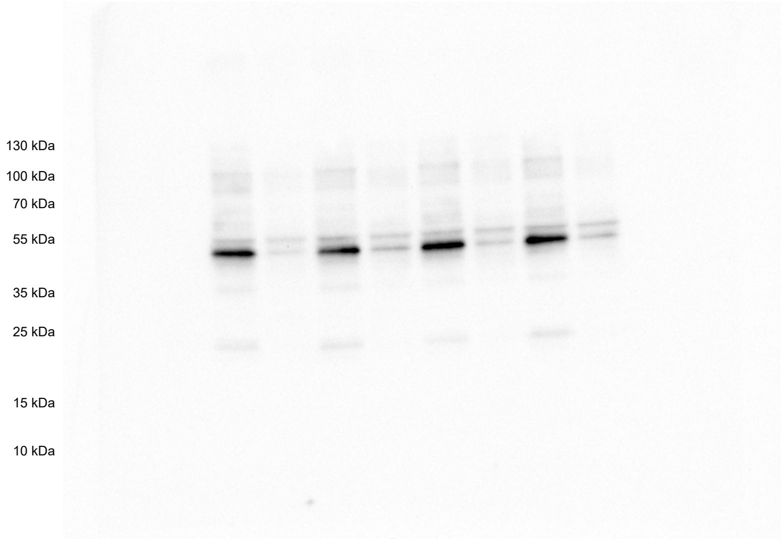

SDMA

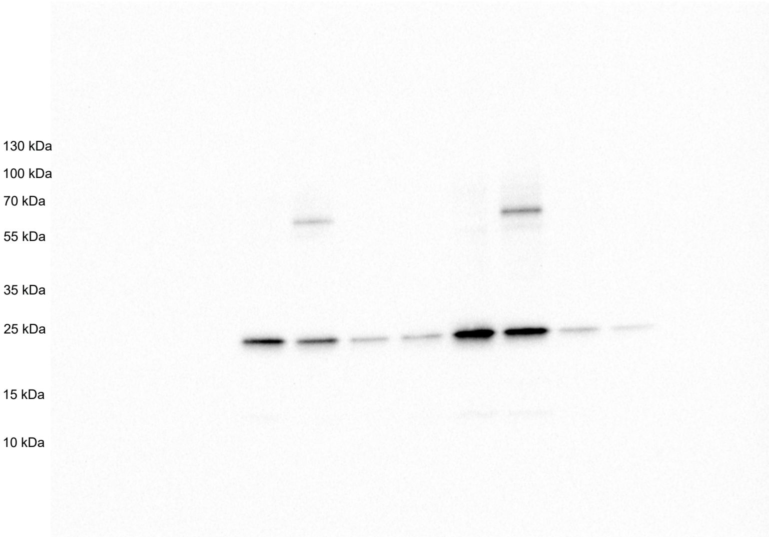

Figure S3B

MMA

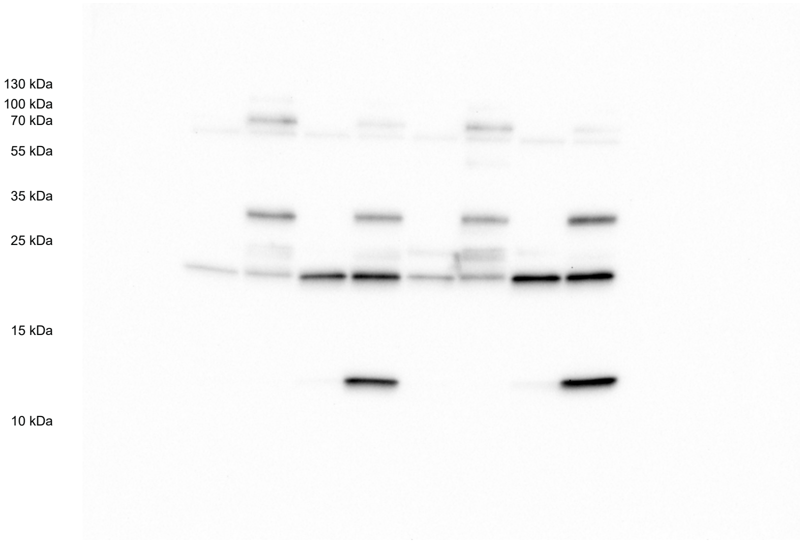

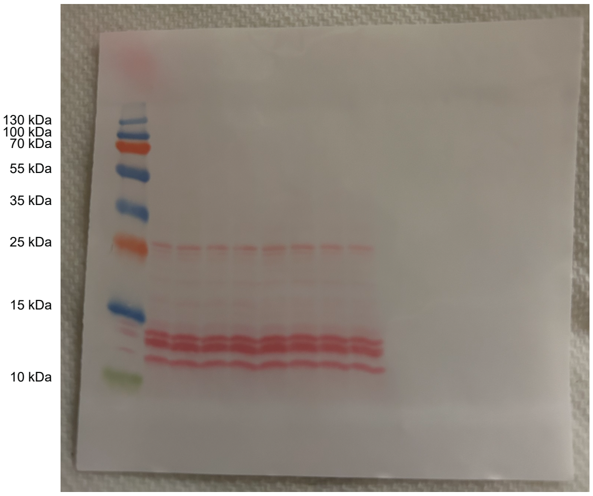

Ponceau

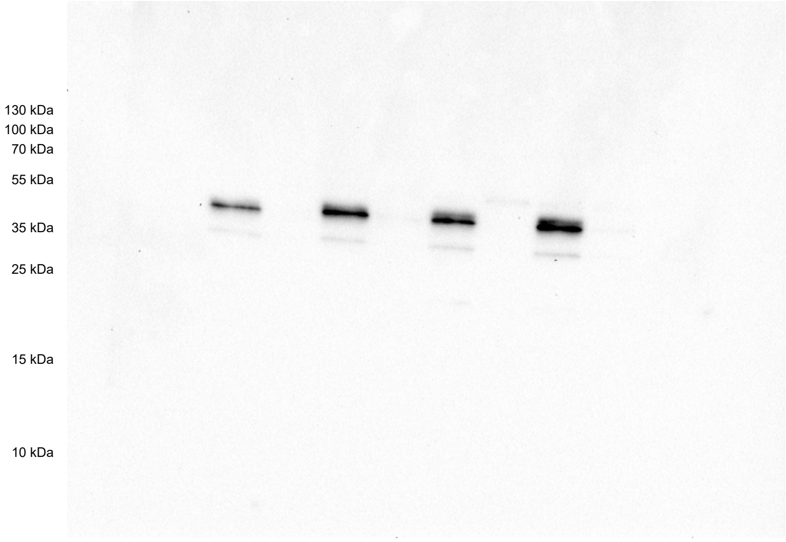

ADMA

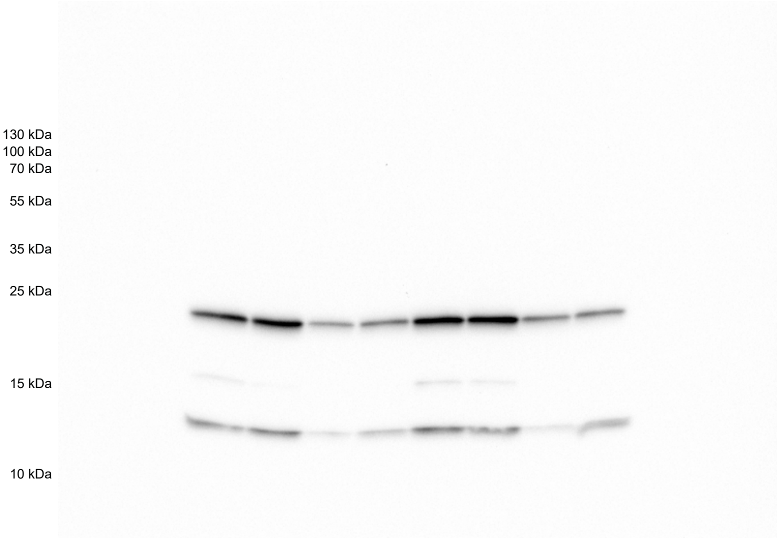

SDMA

Figure S3C

15 kDa

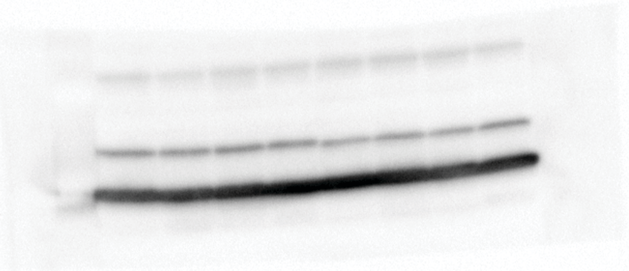

H3

15 kDa

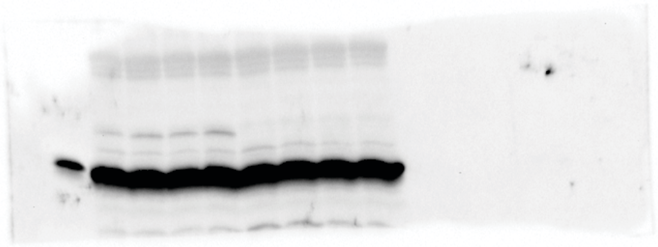

H3K4me3

15 kDa

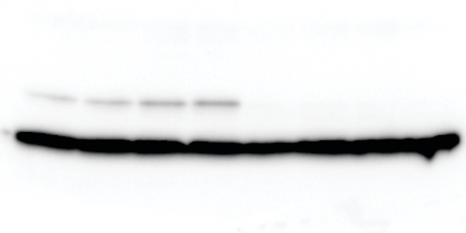

H3K27me3
