# Supplementary material for: Cancer-associated Histone H3 N-terminal arginine mutations disrupt PRC2 activity and impair differentiation
Source: Nat Commun. 2024 Jun 17;15:5155. doi: 10.1038/s41467-024-49486-5 (PMC11183192; doi:10.1038/s41467-024-49486-5)
Supplement: Supplementary file 3 — Description of Additional Supplementary Files [file 41467_2024_49486_MOESM3_ESM.pdf]

Description of Additional Supplementary Files

Supplementary Data 1: Cancer associated histone missense mutations at H3 N-terminal tail arginines. Data are from a previous study[18] and filtered to exclude samples where tumor mutation burden ≥ 10 mutations/Mb.

Supplementary Data 2: Histone posttranslational modification analysis from middle down mass spectrometry.

Supplementary Data 3: Genes differentially expressed in H3 arginine mutants versus H3WT

Supplementary Data 4: H3K27me3 differential peaks

Supplementary Data 5: HA signal at H3K27me3 differential peaks

Supplementary Data 6: H3K4me3 and H3K27me3 differential peak overlaps (H2R2C/H3WT) in MPCs. A hypergeometric test was applied to determine over enrichment in overlap between peak types. Polycomb group and GO term associations are shown in separate tabs.

Supplementary Data 7: Genes with significant expression changes in the same direction in H3R2C and H3R26C versus WT

Supplementary Data 8: Genes associated with GO terms that are significant in a pathway analysis of differentially expressed genes in H3R26C MPCs, or overlapping in H3R2C and H3R26C MPCs, compared to H3WT expressing MPCs.

Supplementary Data 9: Analysis of motifs associated with H3K27me3 loss in H3R26C expressing MPC.

Supplementary Data 10: Gene lists used to calculate module scores.
