# Supplementary material for: Cancer-associated Histone H3 N-terminal arginine mutations disrupt PRC2 activity and impair differentiation
Source: Nat Commun. 2024 Jun 17;15:5155. doi: 10.1038/s41467-024-49486-5 (PMC11183192; doi:10.1038/s41467-024-49486-5)
Supplement: Supplementary file 14 — Reporting Summary [file 41467_2024_49486_MOESM14_ESM.pdf]

nature portfolio

Corresponding author(s): NCOMMS-23-32858

Last updated by author(s): Jun 5, 2024

Reporting Summary

Nature Portfolio wishes to improve the reproducibility of the work that we publish. This form provides structure for consistency and transparency in reporting. For further information on Nature Portfolio policies, see our Editorial Policies and the Editorial Policy Checklist.

Statistics

For all statistical analyses, confirm that the following items are present in the figure legend, table legend, main text, or Methods section.

n/a	Confirmed	
☐	☒	The exact sample size (*n*) for each experimental group/condition, given as a discrete number and unit of measurement
☐	☒	A statement on whether measurements were taken from distinct samples or whether the same sample was measured repeatedly
☐	☒	The statistical test(s) used AND whether they are one- or two-sided<br>*Only common tests should be described solely by name; describe more complex techniques in the Methods section.*
☒	☐	A description of all covariates tested
☐	☒	A description of any assumptions or corrections, such as tests of normality and adjustment for multiple comparisons
☐	☒	A full description of the statistical parameters including central tendency (e.g. means) or other basic estimates (e.g. regression coefficient) AND variation (e.g. standard deviation) or associated estimates of uncertainty (e.g. confidence intervals)
☐	☒	For null hypothesis testing, the test statistic (e.g. *F*, *t*, *r*) with confidence intervals, effect sizes, degrees of freedom and *P* value noted<br>*Give P values as exact values whenever suitable.*
☒	☐	For Bayesian analysis, information on the choice of priors and Markov chain Monte Carlo settings
☒	☐	For hierarchical and complex designs, identification of the appropriate level for tests and full reporting of outcomes
☒	☐	Estimates of effect sizes (e.g. Cohen's *d*, Pearson's *r*), indicating how they were calculated

Our web collection on statistics for biologists contains articles on many of the points above.

Software and code

Policy information about availability of computer code

Data collection	ZEN 2.6 (Blue edition) software was used for image acquisition.
Data analysis	Proteome Discoverer 2.2 (Thermo Fisher Scientific), HistoneCoderTool ProteoformQuant and IsoScale(http://middle-down.github.io/Software/), Rsubread package's align function (version 1.30.6), clusterProfiler (Version 4.0.2), the rtracklayer package (Version 1.40.6), profileplyr (Version 1.12.0), EnhancedVolcano (Version 1.10.0), MEME suite (Version 5.4.1), Salmon (v0.8.1) , ComplexHeatmap R Bioconductor package (v2.8) , DESeq2 R Bioconductor package (v1.32, v.1.20.0) , clusterProfiler R Bioconductor package (v4.0.5), CIS-BP (version 2.0), CellRanger Version 6 , R (4.1.2 and others), Seurat Version 4.2.0, Monocle3 Version 1.3.1, SeuratWrappers Version 0.3.1, and SeuratObject Version 4.1.3 were used in data analysis. Custom code used for analysis is publicly available at https://github.com/soccin/scRNA/tree/proj/p12553 for scRNA-seq analysis and at https://github.com/nacevlab/H3R for all others. Please see the methods section and code availability section for full details.

For manuscripts utilizing custom algorithms or software that are central to the research but not yet described in published literature, software must be made available to editors and reviewers. We strongly encourage code deposition in a community repository (e.g. GitHub). See the Nature Portfolio guidelines for submitting code & software for further information.

Data

Policy information about availability of data

All manuscripts must include a data availability statement. This statement should provide the following information, where applicable:

- Accession codes, unique identifiers, or web links for publicly available datasets
- A description of any restrictions on data availability
- For clinical datasets or third party data, please ensure that the statement adheres to our policy

The sequencing data generated in this study have been deposited in the Gene Expression Omnibus (GEO) database under accession number GSE239638 [https://www.ncbi.nlm.nih.gov/geo/query/acc.cgi?acc=GSE239638]. The mass spectrometry data generated in this study have been deposited in MassIVE database under accession numberMSV000092535 (PXD044141) [https://massive.ucsd.edu/ProteoSAFe/dataset.jsp?task=5f3e25a5d5f34ac598222b7789ab6f94]. Gene lists were obtained from the Molecular Signatures Database using the msigdbr R package (v7.5.1). The CIS-BP database was the source of known motifs (version 2.0). Other source data are provided with this paper as a Source Data file.

Research involving human participants, their data, or biological material

Policy information about studies with human participants or human data. See also policy information about sex, gender (identity/presentation), and sexual orientation and race, ethnicity and racism.

Reporting on sex and gender	N/A
Reporting on race, ethnicity, or other socially relevant groupings	N/A
Population characteristics	N/A
Recruitment	N/A
Ethics oversight	N/A

Note that full information on the approval of the study protocol must also be provided in the manuscript.

Field-specific reporting

Please select the one below that is the best fit for your research. If you are not sure, read the appropriate sections before making your selection.

☒ Life sciences ☐ Behavioural & social sciences ☐ Ecological, evolutionary & environmental sciences

For a reference copy of the document with all sections, see nature.com/documents/nr-reporting-summary-flat.pdf

Life sciences study design

All studies must disclose on these points even when the disclosure is negative.

Sample size	For bulk RNA-seq and CUT&RUN experiments, three independent biologic replicates were analyzed; for sc-RNA-seq one set of data was analyzed.<br>For mass spectrometry experiments two biologic replicates were analyzed. For immunoblotting experiments with cell or histone extracts, the experiment was repeated with two biologic replicates and concordant findings from one representative image was shown. For blotting of recombinant histones, the experiment was performed once under the specific conditions included.<br>For PCR2 assays, five reactions were performed per sample group.<br>For MPC differentiation assays, four biologic replicates were analyzed.<br>For teratoma formation assays, equal number sites were injected for each group; at the end of the experiment, eight WT tumors and seven mutant tumors were available for analysis.<br>No statistical method was used to predetermine the sample size, which was determined based upon prior experience and general practice in the field.
Data exclusions	No data was excluded from the analysis.
Replication	All key findings were reproducible. Please see the figure legends and methods sections for details. Functional differences were assessed by comparing a WT control with a mutant of interest, which bolsters the strength of the conclusions.
Randomization	No randomization was employed since the starting material (i.e., the parental cell line) was similar for all experimental genotypes.
Blinding	Germ layer proportion analysis of the teratomas was performed in a blinded fashion by a board-certified veterinary pathologist. The other experiments, which involved more objective data interpretation and analysis, were not blinded.

Reporting for specific materials, systems and methods

We require information from authors about some types of materials, experimental systems and methods used in many studies. Here, indicate whether each material, system or method listed is relevant to your study. If you are not sure if a list item applies to your research, read the appropriate section before selecting a response.

Materials & experimental systems

n/a	Involved in the study
☐	☒ Antibodies
☐	☒ Eukaryotic cell lines
☐	☐ Palaeontology and archaeology
☐	☒ Animals and other organisms
☐	☐ Clinical data
☐	☐ Dual use research of concern
☐	☐ Plants

Methods

n/a	Involved in the study
☐	☒ ChIP-seq
☒	☐ Flow cytometry
☒	☐ MRI-based neuroimaging

Antibodies

Antibodies used

Experiments using the same antibody (manufacturer, catalog number) were performed over the course of several years and the same lot may not have been used for all experiments. Where available, the lot number used in at least one application is included.

For immunoblotting, the primary antibodies used were: anti-H3 (Abcam, ab1791, lot #s GR3400984, 1041608-1; 1:3000-25000), anti-H3K4me3 (Active Motif, 39159, lot # 314200006, 1:1000), anti-H3K9me3 (Abcam, ab8898, lot # GR3373564-1, 1:1000), anti-H3K27me3 (Cell Signaling, 9733, clone C36B11, lot #s 16, 19; 1:1000 or 1:3500 for the recombinant histone blot), anti-H3K27ac (Active Motif, 39133, 1:1000), anti-H3K27me1 (Active Motif, 61015, clone MABI 0321, 1:1000), anti-H3K27me2 (Cell Signaling, 9728, clone D18C8, 1:1000), anti-H3K4me1 (Abcam, ab8895, 1:1000), anti-H3K4me2 (Abcam, ab7766, 1:1000), anti-HA (Biolegend, 901503, clone 16B12, lot # B242905; 1:1000-5000), anti-SDMA (Cell Signaling, 13222, lot #8; 1:1000), anti-ADMA (Cell Signaling, 13522, lot #5; 1:1000), anti-MMA (Cell Signaling, 8015, lot #7; 1:1000), and anti-beta-actin, (Cell Signaling, 4970, clone 13E5, lot #19; 1:1000). Secondary antibodies: anti-mouse IgG, HRP-linked (Cyvita, NA931, lot # 9648752; 1:5000), anti-rabbit IgG, HRP-linked (Dako, PO399, lot # 20083048; 1:5000), anti-rabbit IgG, HRP-linked (Cell Signaling, 7074, Lot # 32; 1:2000-3000)

For CUT&RUN, the primary antibodies used were: H3 (Abcam, 1791, lot # GR3400984), HA (Biolegend, 901501, clone 16B12, lot # B242905), H3K4me3 (Abcam 39159, lot # 27019006), H3K27me3 (Cell Signaling, 9733, clone C36B11, lot # 17), and rabbit anti-mouse IgG (Abcam, 46450, lot # GR3258049-4). All antibodies were used at a 1:100 dilution.

For nucleosome immunoprecipitation, anti-FLAG-conjugated magnetic beads (Pierce A36797) were used.

For immunofluorescence, the primary antibodies uses were: anti-HA (Biolegend, 901503, clone 16B12; 1:200), total H3 (rabbit ab1791, abcam, 1:1000) and Myosin 4 antibody (Invitrogen, 14650382, clone MF20, lot #2265353, 1 microgram/ml working concentration). Secondary antibodies were AlexaFluor-conjugated secondary antibodies: donkey anti-rabbit AlexaFluor 488 (Invitrogen A21206, 1:1000), goat anti-mouse AlexaFluor 568 (Invitrogen, A11031, 1:1000), anti-mouse AlexaFluor 568 secondary antibody (Invitrogen, A-11004, lot # 2198584, 1:1000).

Validation

For immunoblot experiments, the size of the detected band of interest was confirmed based on a protein marker.
Anti-H3 (Abcam, ab1791): Manufacturer indicates reactivity with human and mouse and suitable applicability in immunoblotting and ChIP.
Anti-H3K4me3 (Active Motif, 39159): Manufacturer indicates reactivity with human and mouse and suitable applicability in immunoblotting and CUT&RUN.
Anti-H3K9me3 (Abcam, ab8898): Manufacturer indicates reactivity with human and mouse and suitable applicability in immunoblotting and ChIP.
Anti-H3K27me3 (Cell Signaling, 9733): Manufacturer indicates reactivity with human and mouse and suitable applicability in immunoblotting and ChIP.
Anti-H3K27ac (Active Motif, 39133): Manufacturer indicates reactivity with human with a predicted wide range of reactivity and suitable applicability in immunoblotting.
Anti-H3K27me1 (Active Motif, 61015): Manufacturer indicates reactivity with human with a predicted wide range of reactivity and suitable applicability in immunoblotting.
Anti-H3K27me2 (Cell Signaling, 9728): Manufacturer indicates reactivity with human and mouse and suitable applicability in immunoblotting.
Anti-H3K4me1 (Abcam, ab8895): Manufacturer indicates reactivity with human and mouse and suitable applicability in immunoblotting.
Anti-H3K4me2 (Abcam, ab7766): Manufacturer indicates reactivity with human and mouse and suitable applicability in immunoblotting.
Anti-HA (Biolegend, 901503): Manufacturer indicates suitable applicability in immunoblotting and immunofluorescence.
Anti-Myosin 4 (Invitrogen 14650382): Manufacturer indicates reactivity with mouse and suitable applicability in immunofluorescence.
Anti-SDMA (Cell Signaling, 13222): Manufacturer indicates reactivity with mouse and suitable applicability in immunoblotting.
Anti-ADMA (Cell Signaling, 13522): Manufacturer indicates reactivity with mouse and suitable applicability in immunoblotting.

	Anti-MMA (Cell Signaling, 8015): Manufacturer indicates reactivity with mouse and suitable applicability in immunoblotting. Anti-beta-actin, (Cell Signaling, 4970):Manufacturer indicates reactivity with mouse and suitable applicability in immunoblotting.

Eukaryotic cell lines

Policy information about cell lines and Sex and Gender in Research

Cell line source(s)	HEK293T and CH310T1/2 were purchased from ATCC. mESCs were previously developed as referenced in the methods section. The sex of the embryos from which the mESCs were derived is unknown based on review of the associated publication (PMID: 25487152).
Authentication	Parental cell lines sourced from ATCC were validated by ATCC. Cell lines were also authenticated based on phenotypes including such as morphology, growth kinetics, differentiation capacity based on information from the cell source and prior experience with these cell lines. Lentiviral constructs used to introduce the mutations of interest confirmed to harbor the correct mutation by Sanger sequencing.
Mycoplasma contamination	Parental cell lines have tested negative for mycoplasma.
Commonly misidentified lines (See ICLAC register)	This study did not use any commonly misidentified cell lines.

Palaeontology and Archaeology

Specimen provenance	*Provide provenance information for specimens and describe permits that were obtained for the work (including the name of the issuing authority, the date of issue, and any identifying information). Permits should encompass collection and, where applicable, export.*
Specimen deposition	*Indicate where the specimens have been deposited to permit free access by other researchers.*
Dating methods	*If new dates are provided, describe how they were obtained (e.g. collection, storage, sample pretreatment and measurement), where they were obtained (i.e. lab name), the calibration program and the protocol for quality assurance OR state that no new dates are provided.*

☐ Tick this box to confirm that the raw and calibrated dates are available in the paper or in Supplementary Information.

Ethics oversight	*Identify the organization(s) that approved or provided guidance on the study protocol, OR state that no ethical approval or guidance was required and explain why not.*

Note that full information on the approval of the study protocol must also be provided in the manuscript.

Animals and other research organisms

Policy information about studies involving animals; ARRIVE guidelines recommended for reporting animal research, and Sex and Gender in Research

Laboratory animals	NOD.Cg-Prkdcscid immunodeficient mice (Jackson Laboratories, Strain 005557), 6–7 week old females.
Wild animals	No wild animals were used in this study.
Reporting on sex	Female mice were used; sex was assigned by the supplier of the animals. Sex was not considered as a variable in this study since our conclusions are based on comparison between different genotypes of allografted mES cells.
Field-collected samples	No field-collected samples were used in this manuscript.
Ethics oversight	Mice were treated in accordance with a protocol approved by the Rockefeller University Institutional Animal Care and Use Committee

Note that full information on the approval of the study protocol must also be provided in the manuscript.

Clinical data

Policy information about clinical studies
All manuscripts should comply with the ICMJE guidelines for publication of clinical research and a completed CONSORT checklist must be included with all submissions.

Clinical trial registration	*Provide the trial registration number from ClinicalTrials.gov or an equivalent agency.*
Study protocol	*Note where the full trial protocol can be accessed OR if not available, explain why.*
Data collection	*Describe the settings and locales of data collection, noting the time periods of recruitment and data collection.*

Outcomes	*Describe how you pre-defined primary and secondary outcome measures and how you assessed these measures.*

Dual use research of concern

Policy information about dual use research of concern

Hazards

Could the accidental, deliberate or reckless misuse of agents or technologies generated in the work, or the application of information presented in the manuscript, pose a threat to:

No	Yes	
☒	☐	Public health
☒	☐	National security
☒	☐	Crops and/or livestock
☒	☐	Ecosystems
☒	☐	Any other significant area

Experiments of concern

Does the work involve any of these experiments of concern:

No	Yes	
☒	☐	Demonstrate how to render a vaccine ineffective
☒	☐	Confer resistance to therapeutically useful antibiotics or antiviral agents
☒	☐	Enhance the virulence of a pathogen or render a nonpathogen virulent
☒	☐	Increase transmissibility of a pathogen
☒	☐	Alter the host range of a pathogen
☒	☐	Enable evasion of diagnostic/detection modalities
☒	☐	Enable the weaponization of a biological agent or toxin
☒	☐	Any other potentially harmful combination of experiments and agents

Plants

Seed stocks	*Report on the source of all seed stocks or other plant material used. If applicable, state the seed stock centre and catalogue number. If plant specimens were collected from the field, describe the collection location, date and sampling procedures.*
Novel plant genotypes	*Describe the methods by which all novel plant genotypes were produced. This includes those generated by transgenic approaches, gene editing, chemical/radiation-based mutagenesis and hybridization. For transgenic lines, describe the transformation method, the number of independent lines analyzed and the generation upon which experiments were performed. For gene-edited lines, describe the editor used, the endogenous sequence targeted for editing, the targeting guide RNA sequence (if applicable) and how the editor was applied.*
Authentication	*Describe any authentication procedures for each seed stock used or novel genotype generated. Describe any experiments used to assess the effect of a mutation and, where applicable, how potential secondary effects (e.g. second site T-DNA insertions, mosiacism, off-target gene editing) were examined.*

ChIP-seq

Data deposition

☒ Confirm that both raw and final processed data have been deposited in a public database such as GEO.

☒ Confirm that you have deposited or provided access to graph files (e.g. BED files) for the called peaks.

Data access links<br>*May remain private before publication.*	GEO accession number: GSE239638<br>Reviewer Token: afqluucgxdgjpsp
Files in database submission	GSE239632_RAW.tar<br>GSE239636_RAW.tar<br>GSE239637_RAW.tar

Genome browser session (e.g. UCSC)

N/A - data visualized with IGV.

Methodology

Replicates

For all samples analyzed by CUT&RUN, we analyzed three biologic replicates.

Sequencing depth

CUT&RUN: Single-end reads, 75 bp.

H3WT, IgG, replicate 1 , total number of reads 6350162
H3WT, IgG, replicate 2 , total number of reads 11583412
H3WT, IgG, replicate 3 , total number of reads 8717526
H3WT, anti-HA, replicate 1 , total number of reads 20526270
H3WT, anti-HA, replicate 2 , total number of reads 108136737
H3WT, anti-HA, replicate 3 , total number of reads 44586212
H3WT, anti-H3K4me3, replicate 1 , total number of reads 7966920
H3WT, anti-H3K4me3, replicate 2 , total number of reads 10891754
H3WT, anti-H3K4me3, replicate 3 , total number of reads 11834161
H3WT, anti-H3K27me3, replicate 1 , total number of reads 9900146
H3WT, anti-H3K27me3, replicate 2 , total number of reads 14383253
H3WT, anti-H3K27me3, replicate 3 , total number of reads 13674371
H3R2C, IgG, replicate 1 , total number of reads 8969240
H3R2C, IgG, replicate 2 , total number of reads 10222995
H3R2C, IgG, replicate 3 , total number of reads 9895032
H3R2C, anti-HA, replicate 1 , total number of reads 18479779
H3R2C, anti-HA, replicate 2 , total number of reads 17200981
H3R2C, anti-HA, replicate 3 , total number of reads 32778112
H3R2C, anti-H3K4me3, replicate 1 , total number of reads 8827376
H3R2C, anti-H3K4me3, replicate 2 , total number of reads 13384055
H3R2C, anti-H3K4me3, replicate 3 , total number of reads 10761794
H3R2C, anti-H3K27me3, replicate 1 , total number of reads 8821814
H3R2C, anti-H3K27me3, replicate 2 , total number of reads 19092251
H3R2C, anti-H3K27me3, replicate 3 , total number of reads 11737985
H3R8C, IgG, replicate 1 , total number of reads 9380436
H3R8C, IgG, replicate 2 , total number of reads 12814451
H3R8C, IgG, replicate 3 , total number of reads 9292011
H3R8C, anti-HA, replicate 1 , total number of reads 13779796
H3R8C, anti-HA, replicate 2 , total number of reads 31620420
H3R8C, anti-HA, replicate 3 , total number of reads 16708077
H3R8C, anti-H3K4me3, replicate 1 , total number of reads 9404379
H3R8C, anti-H3K4me3, replicate 2 , total number of reads 13667332
H3R8C, anti-H3K4me3, replicate 3 , total number of reads 10761794
H3R8C, anti-H3K27me3, replicate 1 , total number of reads 11529161
H3R8C, anti-H3K27me3, replicate 2 , total number of reads 13536053
H3R8C, anti-H3K27me3, replicate 3 , total number of reads 15224091
H3R17C, IgG, replicate 1 , total number of reads 10586524
H3R17C, IgG, replicate 2 , total number of reads 10728314
H3R17C, IgG, replicate 3 , total number of reads 11235519
H3R17C, anti-HA, replicate 1 , total number of reads 10947565
H3R17C, anti-HA, replicate 2 , total number of reads 16756470
H3R17C, anti-HA, replicate 3 , total number of reads 16555521
H3R17C, anti-H3K4me3, replicate 1 , total number of reads 7598943
H3R17C, anti-H3K4me3, replicate 2 , total number of reads 13798340
H3R17C, anti-H3K4me3, replicate 3 , total number of reads 11002292
H3R17C, anti-H3K27me3, replicate 1 , total number of reads 8638386
H3R17C, anti-H3K27me3, replicate 2 , total number of reads 12063500
H3R17C, anti-H3K27me3, replicate 3 , total number of reads 11235519
H3R26C, IgG, replicate 1 , total number of reads 8245658
H3R26C, IgG, replicate 2 , total number of reads 11770326
H3R26C, IgG, replicate 3 , total number of reads 9605221
H3R26C, anti-HA, replicate 1 , total number of reads 10690901
H3R26C, anti-HA, replicate 2 , total number of reads 21313423
H3R26C, anti-HA, replicate 3 , total number of reads 20266652
H3R26C, anti-H3K4me3, replicate 1 , total number of reads 8540455
H3R26C, anti-H3K4me3, replicate 2 , total number of reads 14168175
H3R26C, anti-H3K4me3, replicate 3 , total number of reads 7916545
H3R26C, anti-H3K27me3, replicate 1 , total number of reads 10354038
H3R26C, anti-H3K27me3, replicate 2 , total number of reads 17334044
H3R26C, anti-H3K27me3, replicate 3 , total number of reads 10316931

Antibodies

See the above antibody section and the methods section of the manuscript.

Peak calling parameters

SEACR (version 1.3, stringent, norm 0.01)

Data quality

We manually reviewed tracks with IGV compared to relevant published ChIPseq datasets, checked mapping rates, %RiP and, cross-coverage amongst other QC metrics. Please also see custom code deposited on GitHub.

Software

See methods section and custom code deposited on GitHub.
