# Supplementary material for: Cancer-associated Histone H3 N-terminal arginine mutations disrupt PRC2 activity and impair differentiation
Source: Nat Commun. 2024 Jun 17;15:5155. doi: 10.1038/s41467-024-49486-5 (PMC11183192; doi:10.1038/s41467-024-49486-5)
Supplement: Supplementary file 15 — Source Data [file 41467_2024_49486_MOESM15_ESM.zip › NCOMMS-23-32858_source_data_blots_R4.pdf]

Figure 2A

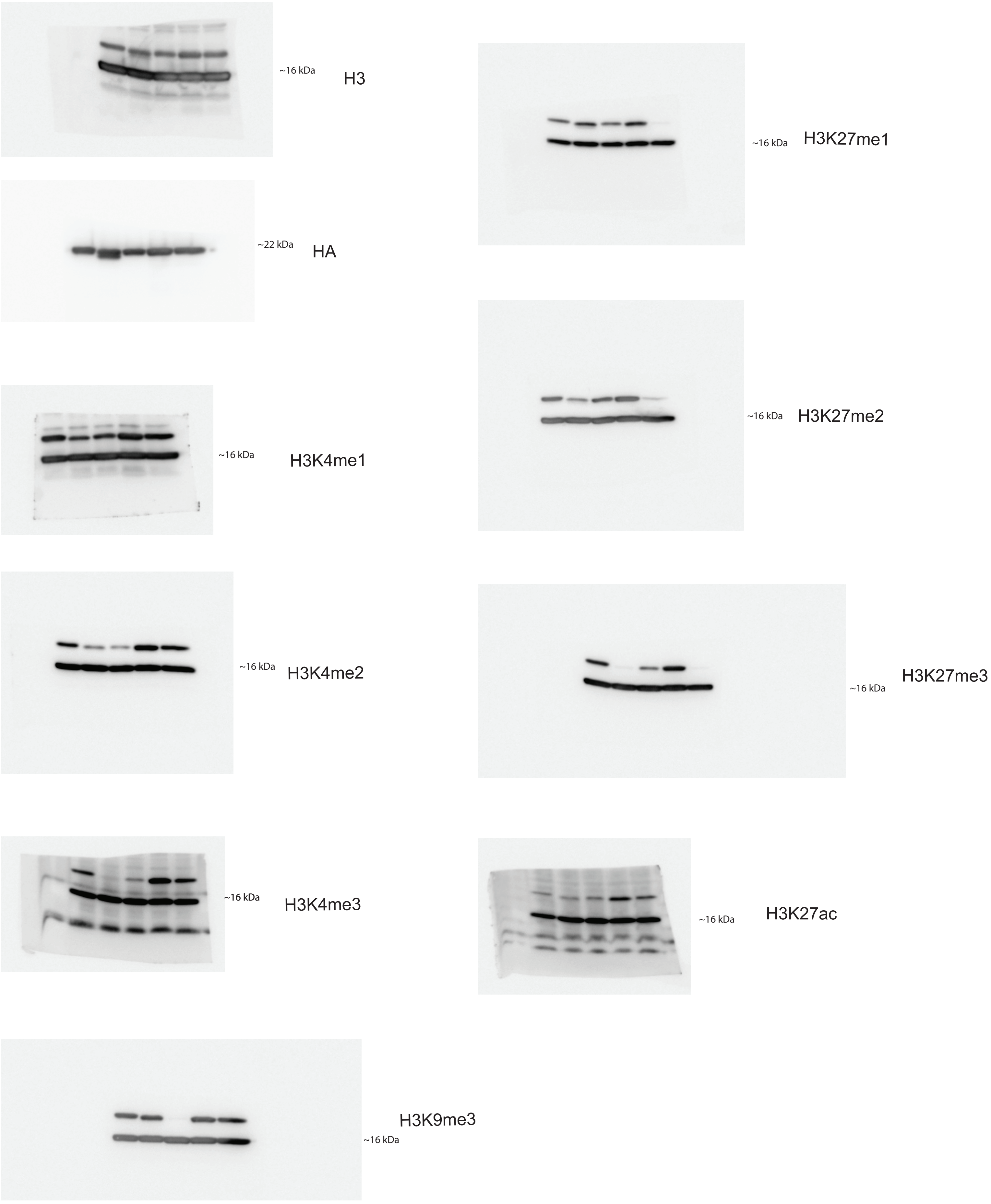
~16 kDa
H3
~22 kDa
HA
~16 kDa
H3K4me1
~16 kDa
H3K4me2
~16 kDa
H3K4me3
~16 kDa
H3K9me3
~16 kDa
H3K27me1
~16 kDa
H3K27me2
~16 kDa
H3K27me3
~16 kDa
H3K27ac

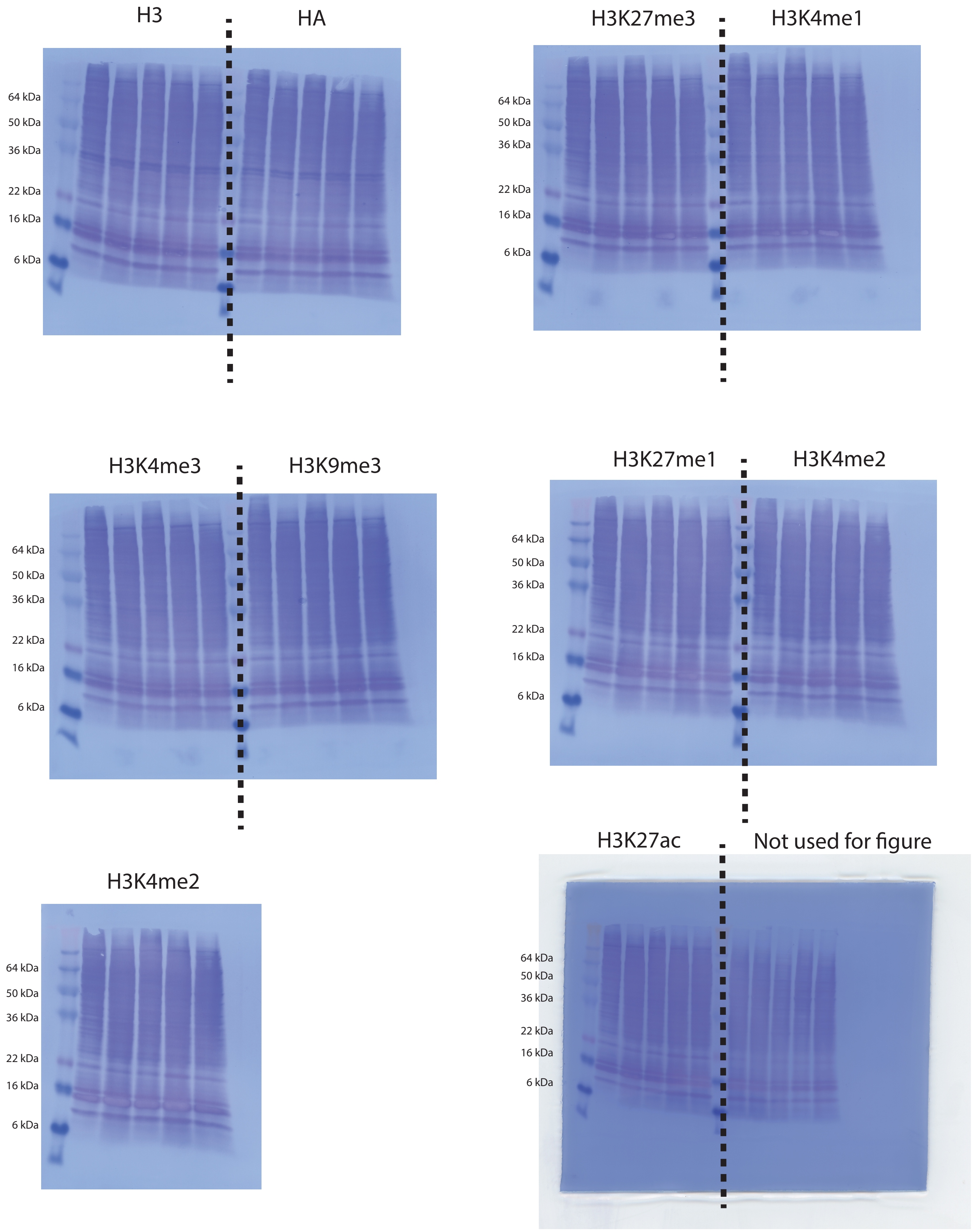
H3
HA
64 kDa
50 kDa
36 kDa
22 kDa
16 kDa
6 kDa
H3K27me3
H3K4me1
64 kDa
50 kDa
36 kDa
22 kDa
16 kDa
6 kDa
H3K4me3
H3K9me3
64 kDa
50 kDa
36 kDa
22 kDa
16 kDa
6 kDa
H3K27me1
H3K4me2
64 kDa
50 kDa
36 kDa
22 kDa
16 kDa
6 kDa
H3K4me2
64 kDa
50 kDa
36 kDa
22 kDa
16 kDa
6 kDa
H3K27ac
Not used for figure
64 kDa
50 kDa
36 kDa
22 kDa
16 kDa
6 kDa

Figure 2B

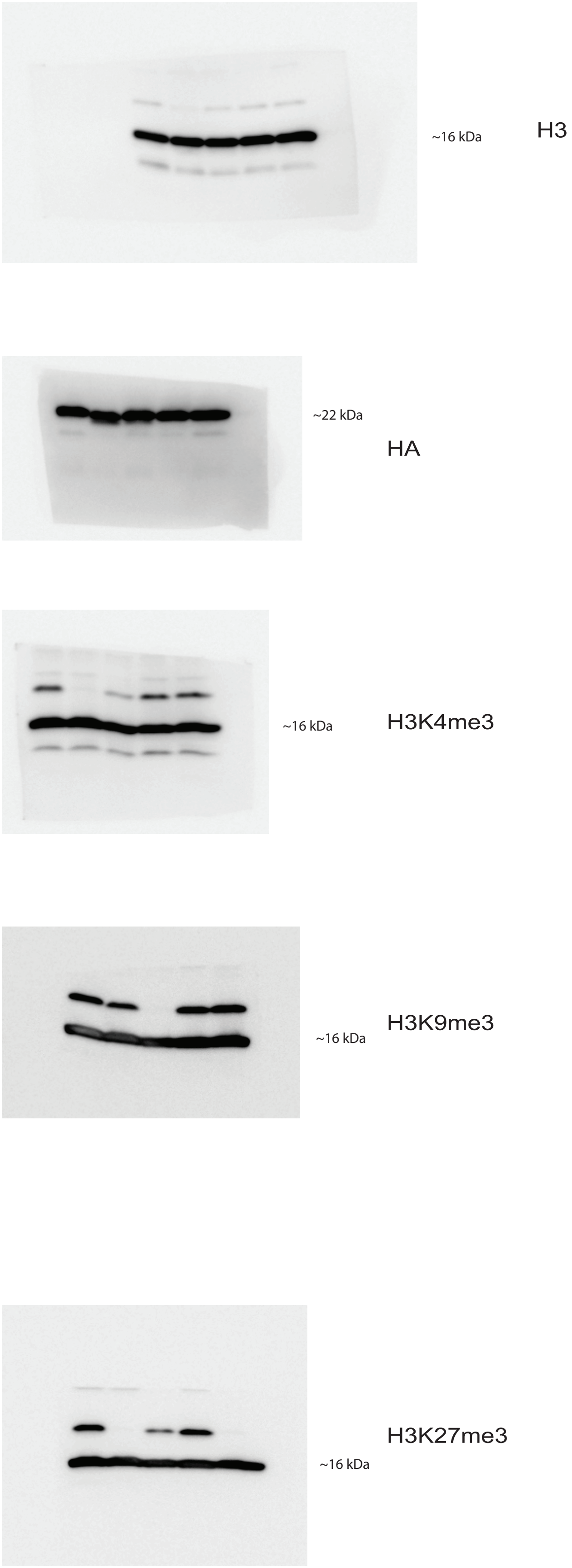
~16 kDa
H3
~22 kDa
HA
~16 kDa
H3K4me3
H3K9me3
~16 kDa
H3K27me3
~16 kDa

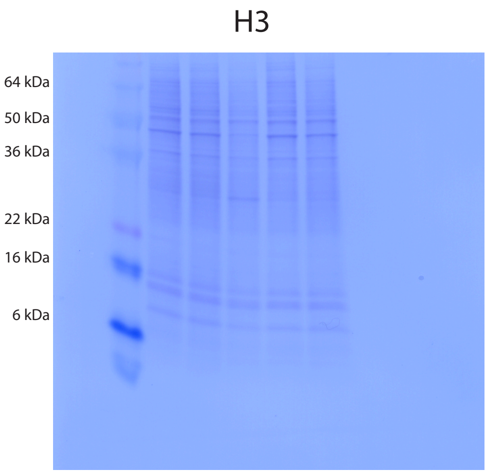
H3
64 kDa
50 kDa
36 kDa
22 kDa
16 kDa
6 kDa

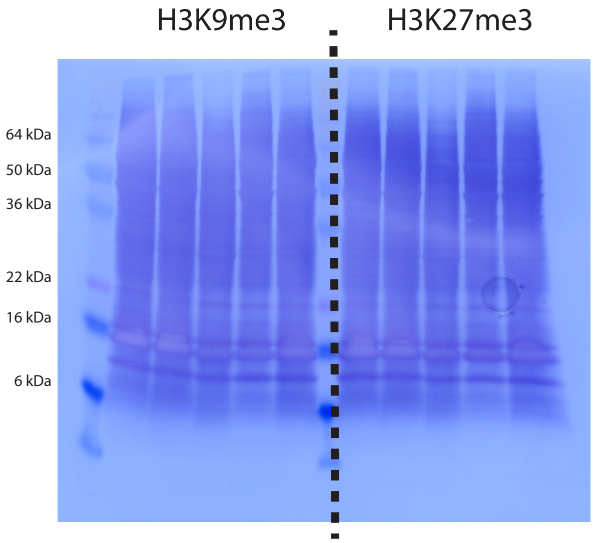
H3K9me3
H3K27me3
64 kDa
50 kDa
36 kDa
22 kDa
16 kDa
6 kDa

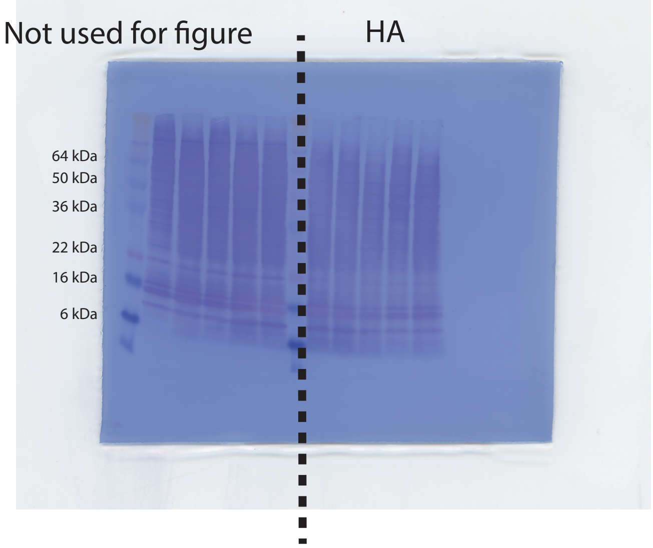
Not used for figure
HA
64 kDa
50 kDa
36 kDa
22 kDa
16 kDa
6 kDa

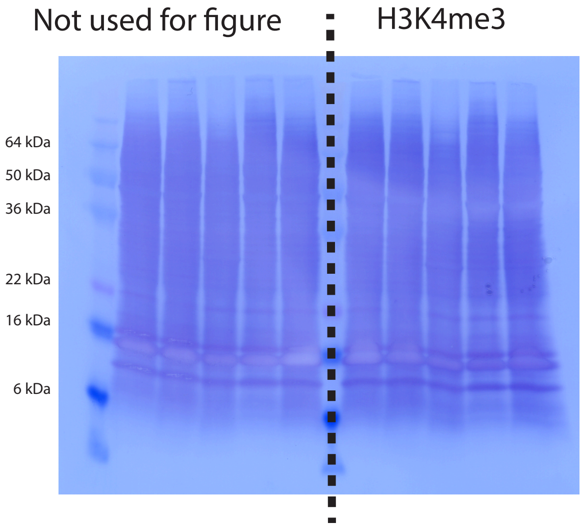
Not used for figure
H3K4me3
64 kDa
50 kDa
36 kDa
22 kDa
16 kDa
6 kDa

Figure 2C

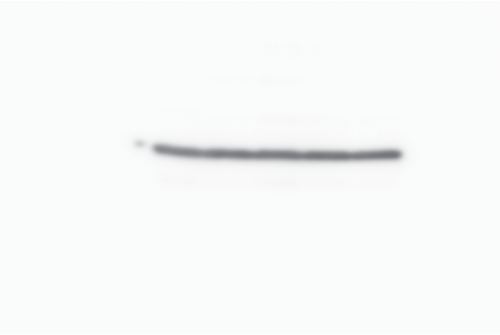

~16 kDa H3

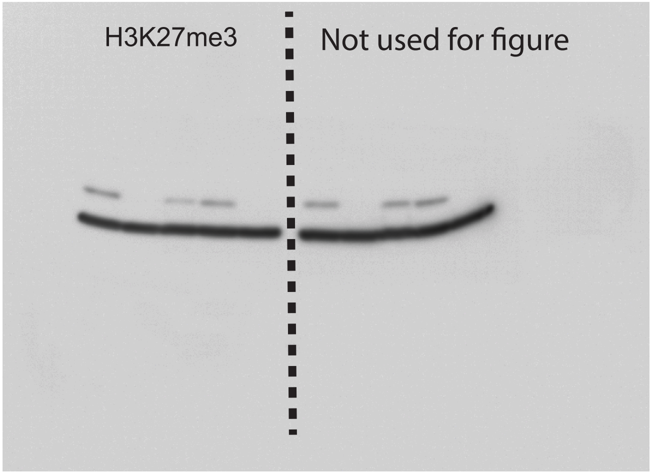

~16 kDa

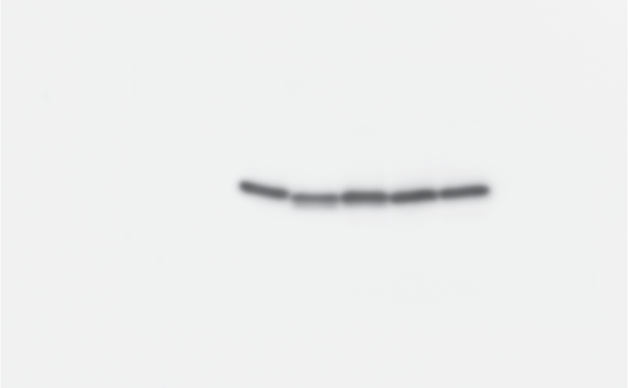

~22 kDa HA

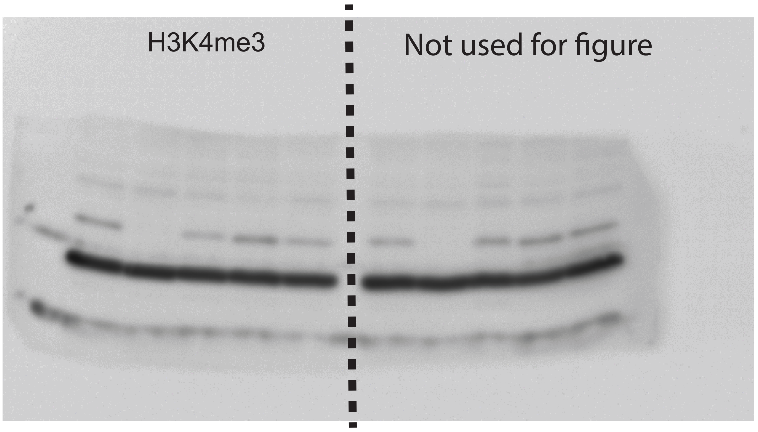

~16 kDa

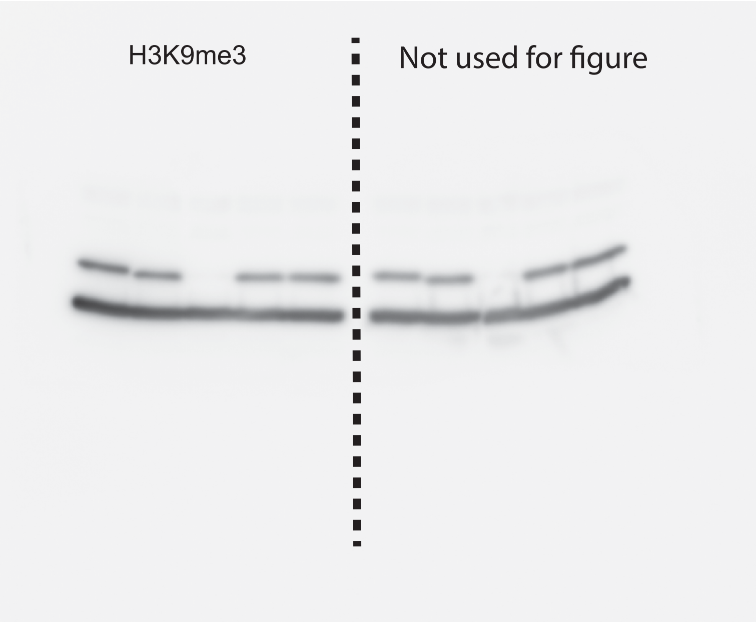

~16 kDa

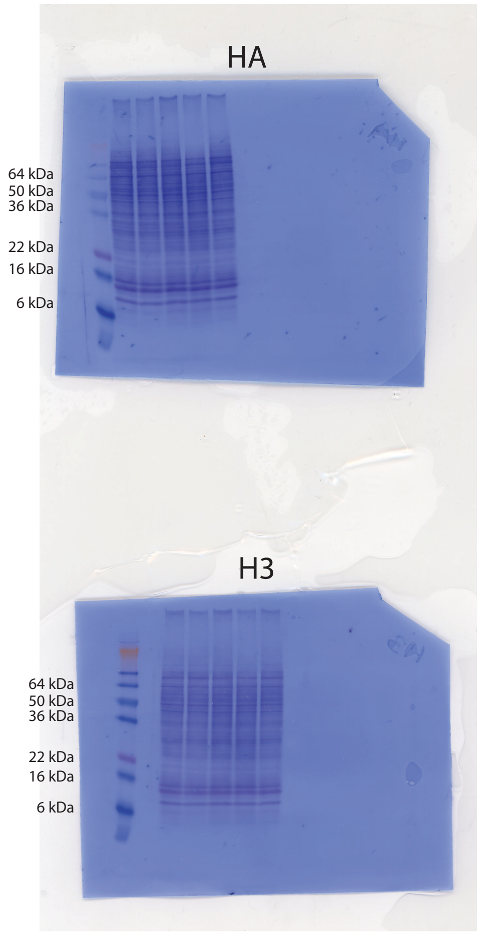
HA
64 kDa
50 kDa
36 kDa
22 kDa
16 kDa
6 kDa
H3
64 kDa
50 kDa
36 kDa
22 kDa
16 kDa
6 kDa

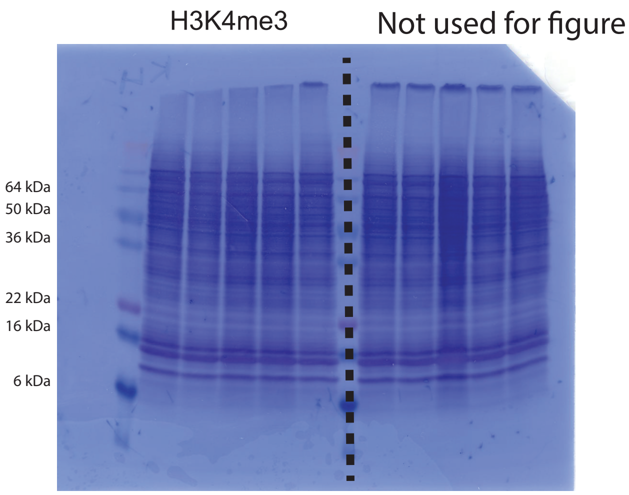
H3K4me3
Not used for figure
64 kDa
50 kDa
36 kDa
22 kDa
16 kDa
6 kDa

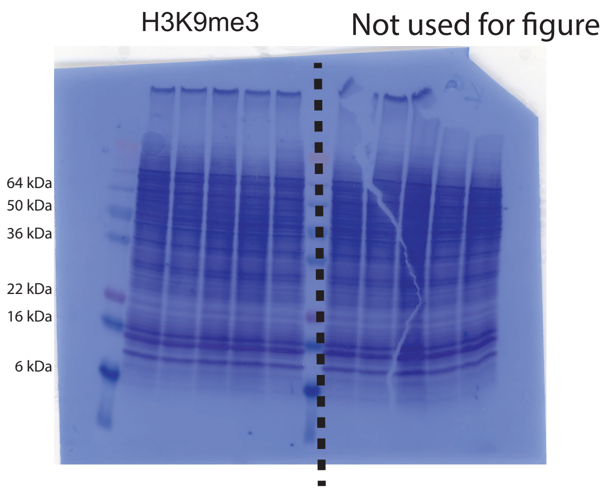
H3K9me3
Not used for figure
64 kDa
50 kDa
36 kDa
22 kDa
16 kDa
6 kDa

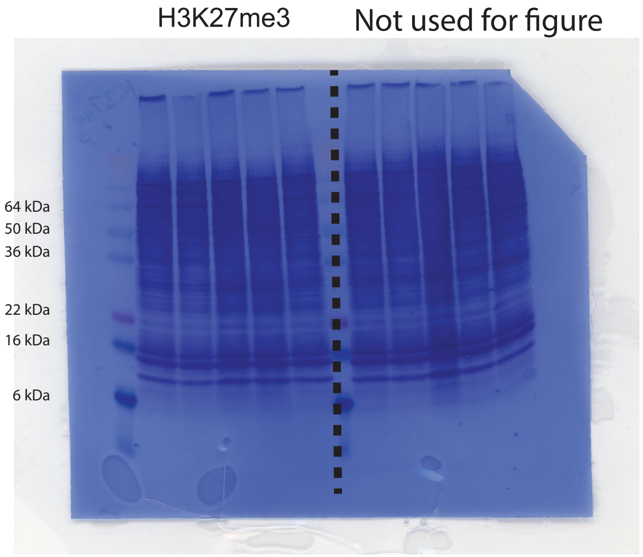
H3K27me3
Not used for figure
64 kDa
50 kDa
36 kDa
22 kDa
16 kDa
6 kDa
